# Multifunctional Gold Nanoparticles for Improved Diagnostic and Therapeutic Applications: A Review

**DOI:** 10.1186/s11671-021-03632-w

**Published:** 2021-12-05

**Authors:** Nicole Remaliah Samantha Sibuyi, Koena Leah Moabelo, Adewale Oluwaseun Fadaka, Samantha Meyer, Martin Opiyo Onani, Abram Madimabe Madiehe, Mervin Meyer

**Affiliations:** 1 Department of Science and Innovation (DSI)/Mintek Nanotechnology Innovation Centre (NIC) Biolabels Node, Department of Biotechnology, University of the Western Cape, Private Bag X17, Bellville, 7535 South Africa; 2 Nanobiotechnology Research Group, Department of Biotechnology, University of the Western Cape, Bellville, South Africa; 3 Department of Biomedical Sciences, Faculty of Health and Wellness Sciences, Cape Peninsula University of Technology, Bellville, South Africa; 4 Organometallics and Nanomaterials, Department of Chemical Sciences, University of the Western Cape, Bellville, South Africa

**Keywords:** Bio-functionalization, Drug delivery, Drug targeting, Gold nanoparticles, Metal-based therapy, Multimodal systems

## Abstract

The medical properties of metals have been explored for centuries in traditional medicine for the treatment of infections and diseases and still practiced to date. Platinum-based drugs are the first class of metal-based drugs to be clinically used as anticancer agents following the approval of cisplatin by the United States Food and Drug Administration (FDA) over 40 years ago. Since then, more metals with health benefits have been approved for clinical trials. Interestingly, when these metals are reduced to metallic nanoparticles, they displayed unique and novel properties that were superior to their bulk counterparts. Gold nanoparticles (AuNPs) are among the FDA-approved metallic nanoparticles and have shown great promise in a variety of roles in medicine. They were used as drug delivery, photothermal (PT), contrast, therapeutic, radiosensitizing, and gene transfection agents. Their biomedical applications are reviewed herein, covering their potential use in disease diagnosis and therapy. Some of the AuNP-based systems that are approved for clinical trials are also discussed, as well as the potential health threats of AuNPs and some strategies that can be used to improve their biocompatibility. The reviewed studies offer proof of principle that AuNP-based systems could potentially be used alone or in combination with the conventional systems to improve their efficacy.

## Introduction

Medicine is among the many fields that have benefitted from nanotechnology. Nanotechnology emerged with a lot of opportunities to improve and develop novel diagnostic and therapeutic agents through the use of nanomaterials [1, 2]. AuNPs, in particular, exhibit unique physicochemical properties and good chemical stability. They are easy to functionalize with almost every type of electron-donating molecules, through various chemistries or based on their strong affinity for thiolated molecules [3, 4]. Due to their tiny size, AuNPs have a larger surface area and high drug loading capacity. Multiple moieties can be incorporated in the AuNPs for biomedical applications; these include targeting molecules to increase specificity, contrast agents for bio-imaging and to monitor disease response to drugs in real time, and therapeutic agents for disease treatment [5, 6]. Interestingly, even without any added biomolecules, AuNPs are capable of targeting, imaging and treatment of diseases. Based on their size-dependent properties, novel AuNP-based systems can be created for use in various biomedical applications [7].

AuNPs are made from a metal precursor that is thermostable and are therefore very stable and non-biodegradable. Bulk gold is used in medicine and had proven to be bio-inert and non-toxic [8, 9]; hence, the gold core in the AuNPs will essentially display similar properties [3, 10]. AuNPs and their applications have been extensively studied for over five decades and have shown great promise as theranostic agents in preclinical [5, 11–13] and clinical studies [14–18]. Many more opportunities for novel AuNP-based systems exist as discussed in this review. AuNPs are already explored in clinical trials as drug carriers for the treatment of late stage cancers [16, 17], and as PT agents in the treatment of prostate cancer [19] and acne [18]. Without undermining the health and regulatory issues that surround the use of AuNPs [20], a future for these systems in biomedicine is at hand. Multifunctional AuNP-based systems that are capable of combating drug resistance with localized and improved efficacy are possible [11, 21, 22]. The review highlights the biological properties of AuNPs in preclinical and clinical studies, by reflecting on their bio-applications as both diagnostic and therapeutic agents. Their potential health threats and strategies that were used to overcome their limitations are also described. Finally, the future perspectives of the AuNPs in medicine are highlighted.

## Gold Nanoparticles

The popularity of AuNPs in medical applications has gained a lot of momentum due to their unique chemical and physical properties. AuNPs are solid colloidal particles that range in size from 1 to 100 nm [23]. The applications of AuNPs in biology are rooted in their physicochemical properties, not limited to their size, surface plasmon resonance (SPR), shape and surface chemistry [3, 10]. These parameters influence their activity and make them perfect candidates for use in disease diagnostics and treatment, either as delivery, sensitizing, contrasts, or therapeutic agents. Their small size is associated with a larger surface area, which allow for surface modification and attachment of multiple payloads, such as targeting, imaging and therapeutic agents [4, 24–26]. Their small size also makes it possible for the NPs and their cargo to cross through biological barriers that are otherwise hard to reach and penetrate [11].

AuNPs are increasingly being recognized as feasible diagnostic, therapeutic and theranostic (an agent that can simultaneously be used to diagnose and treat a disease) agents, which has potential to address the off-target effects associated with conventional therapies. However, AuNPs possess different properties and functions compared with their biocompatible bulk counterparts, which could be hazardous to human health [27–29]. The clinical use of bulk gold compounds for disease treatment is ancient practice and certified as safe [8]. In recent years, research has shown that AuNPs have similar or improved medical properties [29]. Due to their unique optical, chemical and physical properties, AuNPs often present novel properties compared to the bulk gold [30, 31] and can serve as diagnostic and therapeutic agents [5].

### Synthesis of AuNPs

AuNPs can be produced in several ways following either the top-down or the bottom-up approach. The top-down approach uses physical and chemical methods to produce desired sizes from the bulk material, while the bottom-up approach involves chemical methods to assemble the building blocks in the formation of nanosized systems [32, 33]. The physical methods (such as milling, photochemical, radiation and lithography) use extensive energy and pressure to scale down bulk materials into 10^–9^ billionth of a meter in size [10, 32, 34]. Nucleation processes are easily controlled when using the physical methods, reducing agents are not required, and with some of these methods the synthesis occurs simultaneously with the sterilization of the NPs. However, the physical technologies are often costly, not readily available and require specialized equipment. Moreover, capping and stabilizing agents may not survive the high energy processes involved in these processes [34].

**Fig. 1:**
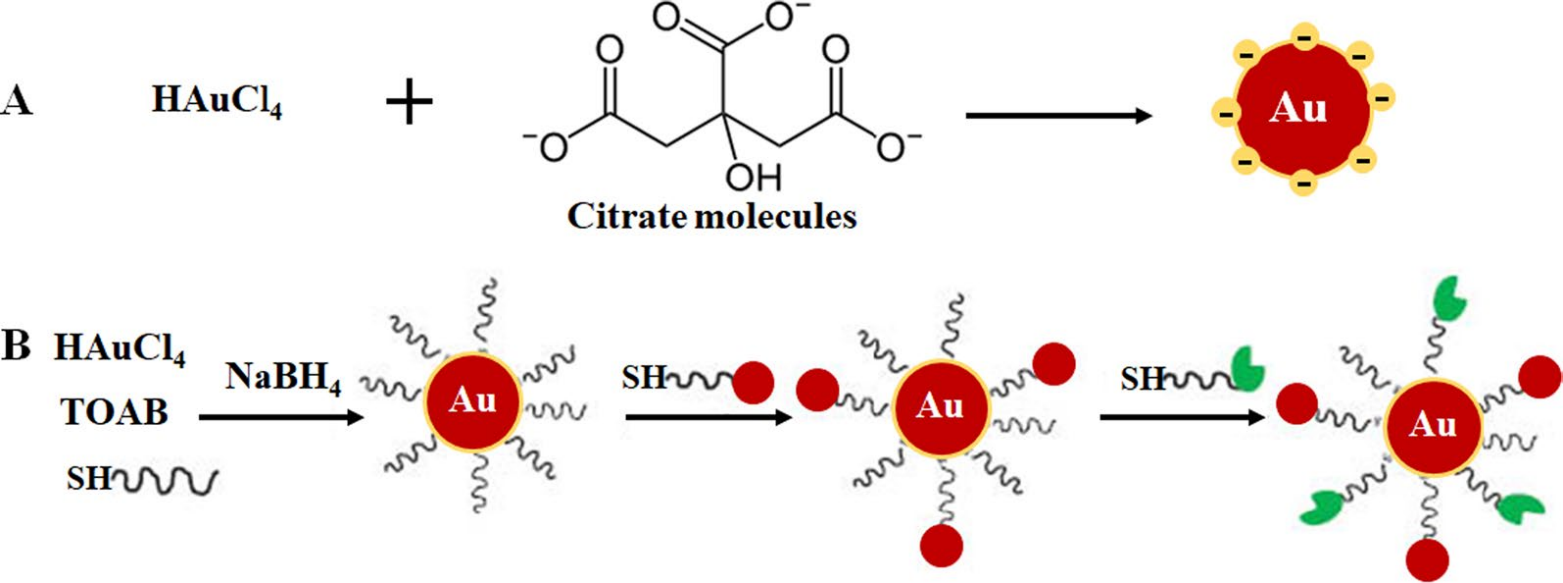
AuNP formulation through one-phase system by citrate reduction (**A**) and two-phase system reduction followed by stabilization and functionalization via ligand exchange reaction, Brust–Schiffrin method (**B**). Reproduced with permission [36]. Copyright 2013, De Gruyter. *TOAB* tetrabutylammonium bromide, *SH* thiolated molecules

The bottom-up approach is mostly preferred in the synthesis of AuNPs as it is rapid, is easy and does not require the use of sophisticated equipment [33–35]. It is based on the chemical method developed by Turkevich in 1951 (Fig. 1A), which use citrate for reduction and stabilization of a gold precursor, resulting in the production of 15-nm spherical AuNPs [3, 10, 23, 33, 36, 37]. The method was further modified by varying the ratio of citrate to gold precursor content and resulted in size diameter range of 15–150 nm AuNPs (Fig. 1B) [10, 24]. A number of reducing agents such as sodium borohydride, cetyltrimethylammonium bromide (CTAB) and ascorbic acid were also introduced. Some of the chemical reducing agents are unfortunately toxic [33, 34, 36] and usually passivated by adding stabilizing agents on their surface such as polyethylene glycol (PEG), gum arabic, polysaccharides and bio-active peptides [37, 38].

Greener approaches such as microwave-induced plasma-in-liquid process (MWPLP) and green nanotechnology have been explored in synthesis AuNPs to avoid the use of toxic chemical reducing agents. The MWPLP uses microwaves to generate nucleation of metallic NPs and does not require any reducing agents, and the energy required for the synthesis is very low [34]. Green nanotechnology, on the other hand, uses natural compounds originating from plants and microorganisms as a source of reducing agents in the synthesis of biogenic AuNPs [12, 33, 39–41]. Green nanotechnology is considered as eco and environmentally friendly and thus more suitable for biomedical applications. Plant-mediated synthesis is more economical than using microorganisms. Moreover, the synthesis can be performed in just one step, and the NPs are easier to purify. In addition, plants are renewable; various parts of the plants such as leaves, stems, barks, roots, flowers and fruits can be harvested without killing the plant and used for synthesis. Extracts prepared from the plant material contain phytochemicals, proteins and enzymes that can function as the reducing, stabilizing and capping agents [10, 12, 24, 34, 35, 40, 42]. Epigallocatechin from green teas [42] and mangiferin (MGF) from mangoes [12, 43] are among plant-derived compounds that have been extensively used to synthesize AuNPs [34]. More information on these methods is extensively reviewed in the following references [10, 24, 34, 35].

## Biological Application of AuNPs

The role and significance of AuNPs in medical science are undoubtedly becoming more visible, which is backed by the increasing number of studies demonstrating their multifaceted application in a wide range of biomedical fields. The biocompatibility of AuNPs is attributed to the long history of gold in the treatment of human diseases, which goes back to 2500–2600 BC. Chinese and Indian people used gold for the treatment of male impotence, epilepsy, syphilis, rheumatic diseases and tuberculosis. China discovered the longevity effect of red colloidal gold, which is still practiced in India as part of Ayurvedic medicine for rejuvenation and revitalization. Cinnabar-gold (also known as Makaradhwaja) is used for improved fertility in India. In the Western countries, gold has been used to treat nervous disorder and epilepsy. No toxicity was reported for its use in both in vitro and in vivo studies [8, 44, 45]. Since then, oral and injectable gold compounds continued to be used as treatments for arthritis [9, 46] and have also been shown to have anticancer effects [8]. Similar and in some instances improved effects were also reported for AuNPs, which are emerging as promising agents for disease diagnosis [47–49] and therapy [3, 29, 50, 51].

**Fig. 2:**
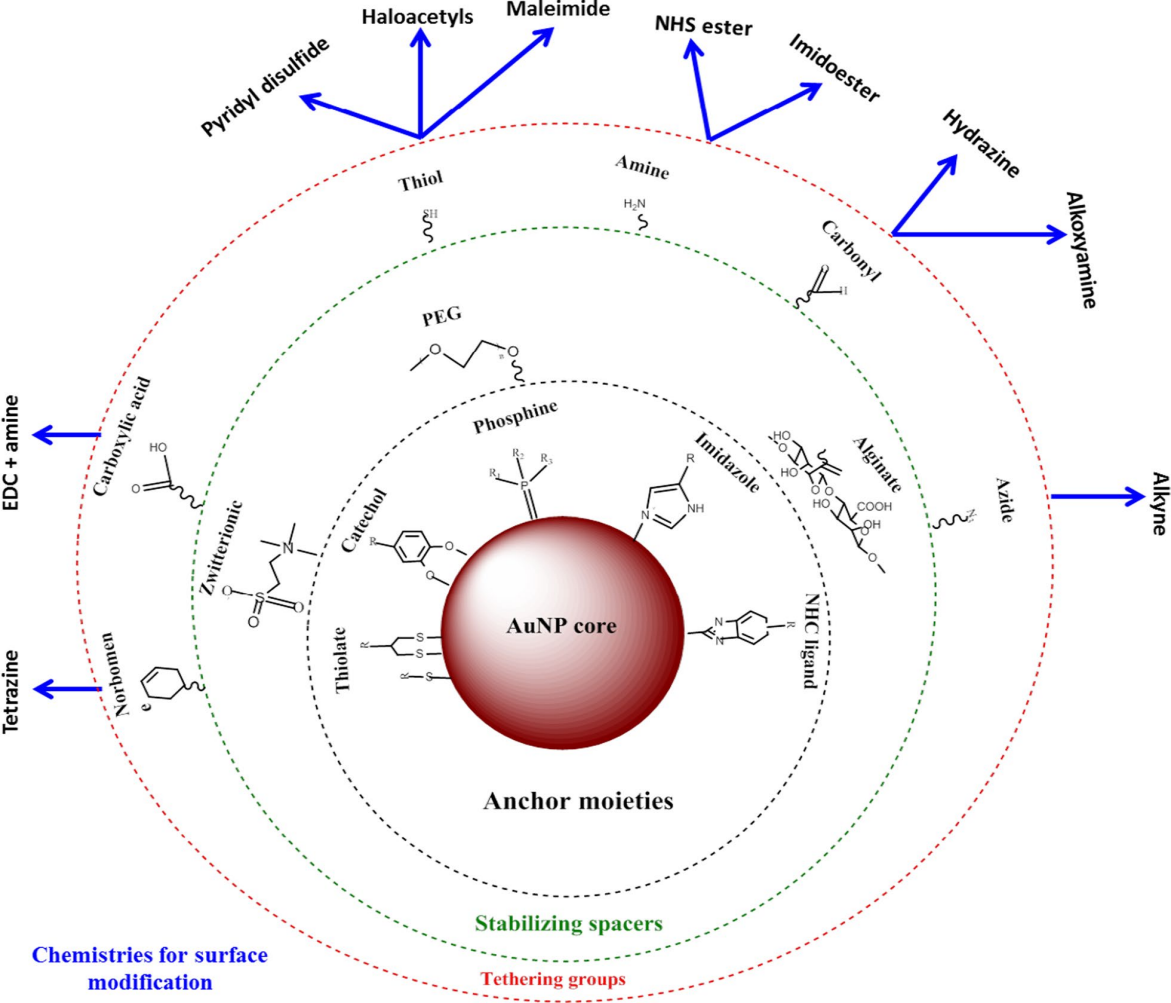
Synthesis and functionalization of AuNPs. Biomolecules with functional groups are first adsorbed on the NP surface through gold-thiol affinity. Then, other functional groups such as amine group can be used to bind molecules with a carboxyl groups to attach targeting or drug moieties. Adapted from [32]

AuNPs have a larger surface area that can be exploited for biomedical applications, by attaching various biomolecules to suit a desired function. These can include targeting moieties to help recognize disease-specific biomarkers, contrasts agents for bio-imaging and therapeutic agents for treatment of diseases [24, 25]. The advantage of using AuNPs over other nanomaterials is that they can be easily functionalized using various chemistries as demonstrated in Fig. 2 [4, 26]. AuNPs have high affinity for thiolated molecules, and thiol-gold binding is the most commonly used method to adsorb molecules onto the NP surface [4]. Affinity-based chemistries such as biotin-streptavidin binding and carbodiimide coupling are also used. AuNPs are used in three main areas of biomedicine: delivery of pharmaceuticals, diagnostic and therapeutic purposes [24, 35], and have demonstrated a huge potential in these areas as discussed below.

### AuNPs as Drug Delivery Agents

The most common application of AuNPs is as delivery vehicles for drugs [11, 18, 52], vaccines [53] and gene therapy [24, 32]. AuNPs possess properties that can resolve most of the issues associated with conventional therapies such as drug resistance, low drug distribution, biodegradation and early drug clearance [11]. AuNPs can significantly reduce drug dosage, treatment frequency and capable of transporting hydrophobic and insoluble drugs. They are considered to be bio-inert and can mask their cargo from attack by immune cells, protect the drugs from proteolytic degradation as they travel through the circulatory system, and thus increase the drug circulation time. These factors can readily increase the efficacy of the drugs by concentrating and retaining them in the diseased tissues with little or no effect to the normal tissues [25].

The use of AuNPs in cancer treatment has been extensively studied [17, 37, 54], and over the years it has been extended to other diseases such as obesity [50, 55, 56] and acne [18]. Nano-based systems are smaller than most cellular components and can passively transverse through cellular barriers by taking advantage of the enhanced permeability and retention (EPR) effect on the vasculature of the diseased tissues [25]. The EPR in a pathological state is characterized by excessive angiogenesis and increased secretion of permeability mediators, which can enhance AuNP uptake by the diseased tissues. These characteristics are only associated with pathological states and not normal tissues, which provide an opportunity for selective targeting of the AuNP conjugates [25]. AuNPs are attractive as drug carriers as they can carry multiple molecules simultaneously, further diversifying their properties. This is a desirable trait in medicine in which most of the AuNP bio-applications are rooted upon, as AuNPs can be tailored for a specific biomedical function. This can help control the way they interact with cellular organelles and therefore hold promise for future development of effective diagnostic and treatment modalities for various diseases [4].

### AuNP-Based Diagnostic Systems

The emergence of nanotechnology has raised the stance in developing detection systems that are rapid, robust, sensitive and highly competitive compared to the conventional diagnostic tests [48]. Nanomaterials are usually integrated in existing biosensing platforms for the detection of gases, DNA and protein markers involved in the development of diseases [47]. Among the various nanomaterials (which include metallic, polymeric, magnetic and semiconductor NPs) used in diagnostics, AuNPs have been widely used in biosensors, electrochemical sensors and chromogenic assays to detect or sense the presence of disease biomarkers [49]. Their localized SPR (LSPR), fluorescence resonance energy transfer (FRET), surface-enhanced Raman scattering, conductivity, redox activity and quantized charging effect make them an ideal tool for imaging and detection of target molecules [10, 24]. Their electronic and optical properties, and ability to scatter visible and near-infrared (NIR) light are compatible and measurable with various technologies such as microscopic techniques (electron, confocal and dark-field light scattering) [57], computed tomography (CT), PT heterodyne imaging technique, UV–Vis and Raman spectroscopy [24, 35].

The development of AuNP-based diagnostic systems involves modification of the AuNP surface, for example, through the attachment of biomolecules that recognize disease biomarkers [3, 24, 58]. Lateral flow assays (LFAs) are probably the best-known example of nanotechnology-based diagnostic tools. LFAs typically make use of AuNPs of about 30–40 nm because smaller particles have a very small extinction cross sections, whereas larger particles are usually unstable for use in these assays [59]. In addition, other molecules/enzymes that can trigger changes in SPR, conductivity and redox of AuNPs are included. These indicators give a detectable signal after binding of analytes to the AuNP conjugates [24], lack or presence of signal will then reflect the absence or presence of the target molecule or the disease. The signal generated by AuNPs is chemically stable, long-lasting and consistent when used in different test formats: test tube, strip, in vitro and in vivo [24]. Hence, their application has remarkably increased the speed and success of diagnostic assays.

#### Colorimetric AuNP-Based Assays

In colorimetric assays, AuNPs produce a visual signal (usually a color change) that can be detected with the naked eye without the use of advanced instruments. Generally, a colloidal solution of AuNPs has a ruby red to grape color that is highly dependent on the interparticle distance [60, 61]. Binding of an analyte to the AuNPs modified with molecular bio-recognition elements (e.g., antibodies, peptides, aptamers, enzymes, etc.) induces a distinct shift in the LSPR, consequently resulting in the change of color from ruby red to blue [60, 62, 63]. The intensity of color is directly proportional to the concentration of an analyte and used to confirm the presence and state of the disease. The AuNP-based colorimetric diagnostics has been used successfully in the detection of influenza A virus [64], Zika virus [65], T7 Bacteriophage [66], *Mycobacterium tuberculosis* [67], and recently, for the detection of severe acute respiratory syndrome-coronavirus-2 (SARS-CoV-2) [60, 68].

**Fig. 3:**
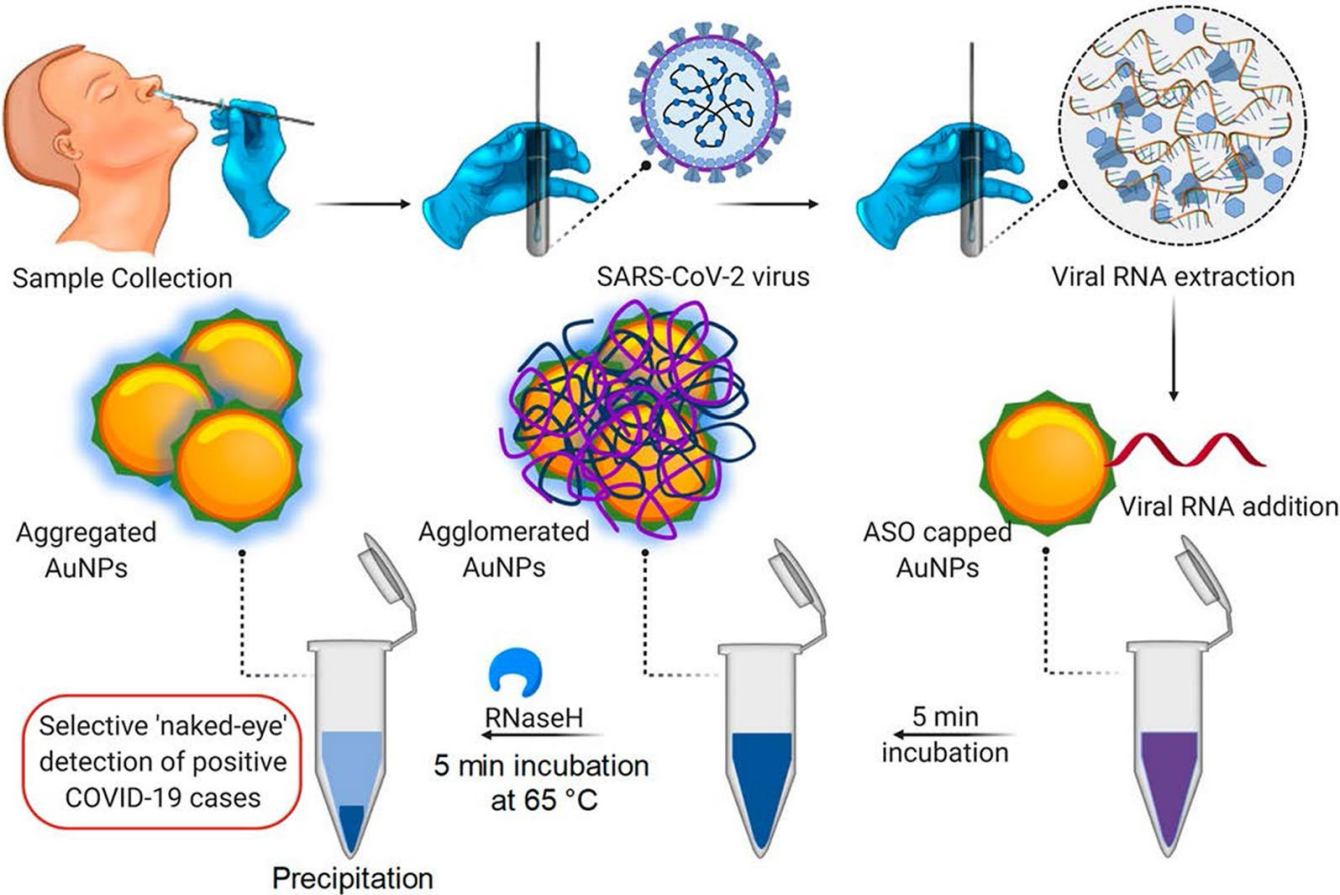
AuNP-based colorimetric diagnostic system. Selective naked eye detection of SARS-CoV-2 RNA by the ASO-capped AuNPs. Reproduced with permission [60]. Copyright 2020, ACS Nano

An example of a colorimetric AuNP-based assay was demonstrated for the detection of SARS-CoV-2 [60], a virus that causes a highly infectious Corona virus disease 2019 (COVID-19) [60, 68]. With this assay, the presence of the virus was reported by a simple color change; no instrumentation was required to do the diagnosis. The current clinical diagnostic tests of this virus either use the reverse transcriptase real-time polymerase chain reaction (RT-PCR) assay, which takes 4–6 h, while the rapid point-of-care (PoC) systems detect antibodies that might take several days to appear in the blood. In comparison, the colorimetric AuNP-based assay was more robust and faster as demonstrated in Fig. 3. Incubating AuNPs-tagged with antisense oligonucleotides (ASOs) in the presence of SARS-CoV-2 RNA samples resulted in the formation of blue precipitate within ~ 10 min. In a SARS-CoV-2-positive test, binding of the ASOs to the N-gene in the nucleocapsid phosphoprotein of the virus induced a blue color that was visually detected. The test was very sensitive and had a limit of detection of 0.18 ng/μL for the SARS-CoV-2 RNA [60].

**Fig. 4:**
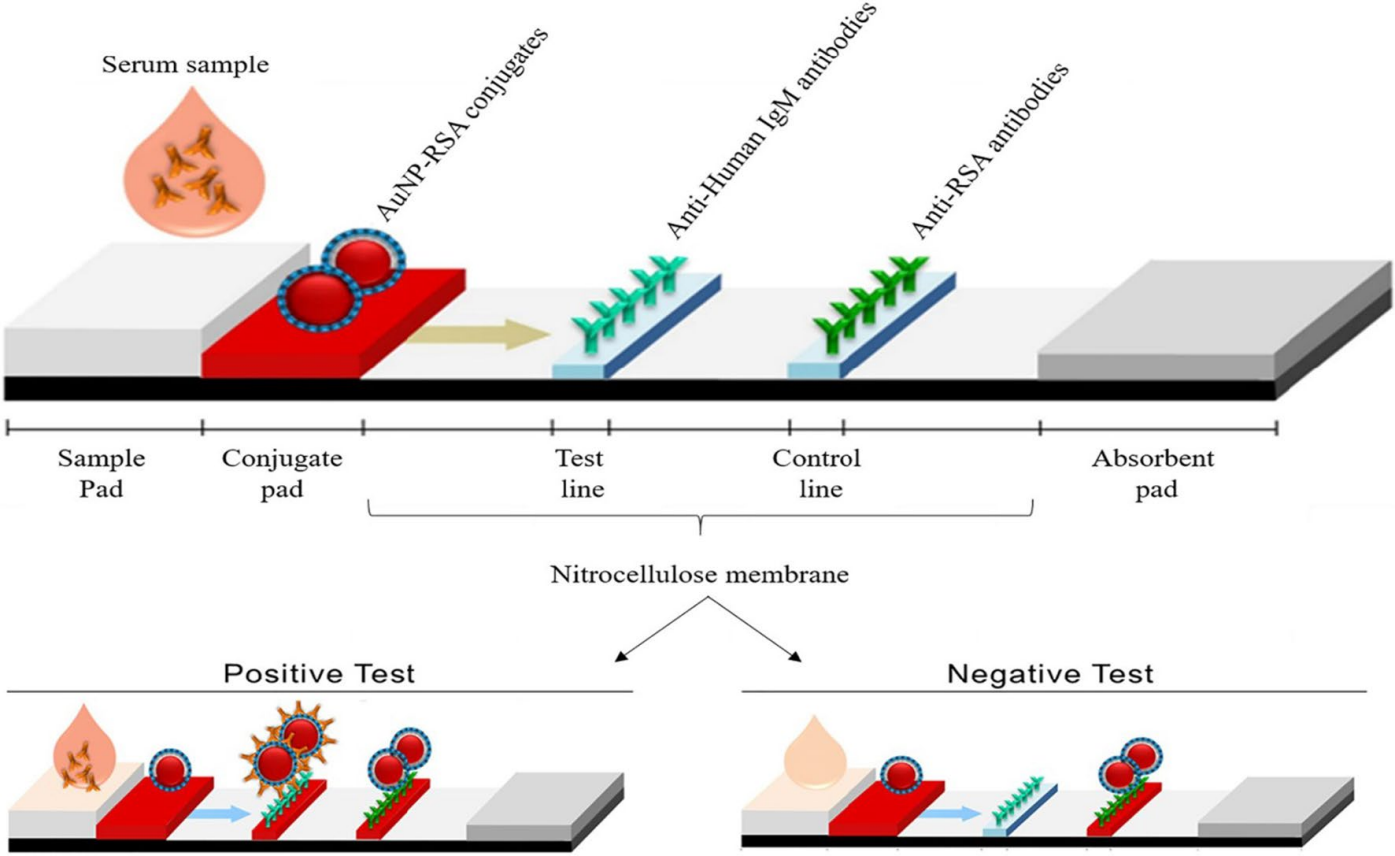
AuNP-based LFAs for detection of IgM *P. jirovecii* antibodies. The presence (positive test) or absence (negative control) of the *P. jirovecii* antibodies could be differentiated by the AuNP reddish color in both test and control lines, or only in the control line, respectively. Reproduced with permission [70]. Copyright 2019, Frontiers in Microbiology

AuNP-based LFAs follow the same principle as the one shown in Fig. 3; however, instead of a color change in a solution, a visible line is formed on a test strip when an analyte is present. In the presence of an analyte, AuNPs were captured on the test line and formed a distinct red line, which was visualized by the naked eye. The intensity of the line is determined by the number of adsorbed AuNPs [69]. An example of a simple and rapid AuNP-based LFA is shown in Fig. 4, for the detection of *Pneumocystis jirovecii* (*P. jirovecii*) IgM antibodies in human sera. The 40 nm AuNPs were conjugated with the recombinant synthetic antigens (RSA) of *P. jirovecii*, either the major surface glycoprotein or kexin-like serine protease, which were used as an indicator for the presence or absence of *P. jirovecii*. In a positive test, the *P. jirovecii* IgM was captured by the AuNP-RSA conjugate at the conjugate pad. The AuNP-RSA/IgM complex then flows to the analytical membrane where it binds the anti-human IgM (test line) and the excess move to the anti-RSA antibodies (control line), resulting in two red lines. The negative test will only have a red color on the control line [70]. An independent study used the AuNP-based LFA to selectively detect the SARS-CoV-2 IgM as confirmed by the appearance of the red lines in both test and control lines [68]. The color was visually detected by naked eyes within 15 min in the two systems, and only 10–20 μL serum samples were needed per test [68, 70].

One of the first examples of the use of AuNPs as a signaling probe on a LFA was for the detection of Ramos cells; the TE02 aptamer was used as a capture probe and the TD05 aptamer as a detection probe. The aptamer-AuNP biosensor can visually detect a minimum of 4 000 Ramos cells without any instrumentation and 800 Ramos cells with a portable strip reader within 15 min. Using this sandwich detection biosensor, the assay successfully detected Ramos cells spiked in human blood [71] and was used as a proof of concept for developing a rapid, sensitive and low-cost systems for qualitative and quantitative detection of circulating cancer cells. Since then, various AuNP-based LFAs have been designed for the diagnosis of numerous infectious diseases, including diseases caused by *Pneumocystis pneumonia* [70], Ebola virus [72], HIV, Hepatitis C virus, and *Mycobacterium tuberculosis* [73] and more recently SARS-CoV-2 virus [68].

#### AuNP-Based Imaging Systems

AuNPs have been intensively investigated for applications in bio-imaging because of their ability to absorb and scatter light matching their resonance wavelengths, up to 10^5^ times more than the conventional fluorophores [74]. AuNPs have a higher atomic number and electron density (79 and 19.32 g/cm^3^) as compared to the conventional iodine-based agents (53 and 4.9 g/cm^3^), thus proving to be better contrast agents [24]. The AuNPs amass on the diseased cells or tissues and induce a strong X-ray attenuation making the targeted site highly distinct and easily detectable. AuNPs are attached to chemical moieties and molecular bio-recognition agents that can selectively target specific antigens to induce distinct and target-specific contrast for CT imaging [75].

In vitro targeted molecular CT imaging system was achieved by using AuNPs functionalized with a RNA aptamer that binds to the prostate-specific membrane antigen (PSMA). The AuNP–PSMA aptamer conjugate showed more than fourfold CT intensity for the PSMA-expressing prostate (LNCaP) cells compared to the PC-3 prostate cells, which lacks the target receptor [76]. Similarly, AuNP-diatrizoic acid-AS1411 aptamer conjugate was localized in CL1-5 (human lung adenocarcinoma) cells and CL1-5 tumor-bearing mice. The AS1411 aptamer targets nucleolin (NCL) receptor that is expressed by the CL1-5 cells on the cell surface, while diatrizoic acid is an iodine-based contrast agent. The AuNP–diatrizoic acid–AS1411 aptamer conjugate had a linear attenuation curve with a slope of 0.027 mM Au Hounsfield unit (HU^−1^) indicating accumulation of the AuNPs at the tumor site [77]. The AuNPs exhibited a longer vascular retention time, which prolonged their circulation time in the blood [77–79] and improved the CT signal of diatrizoic acid [77].

**Fig. 5:**
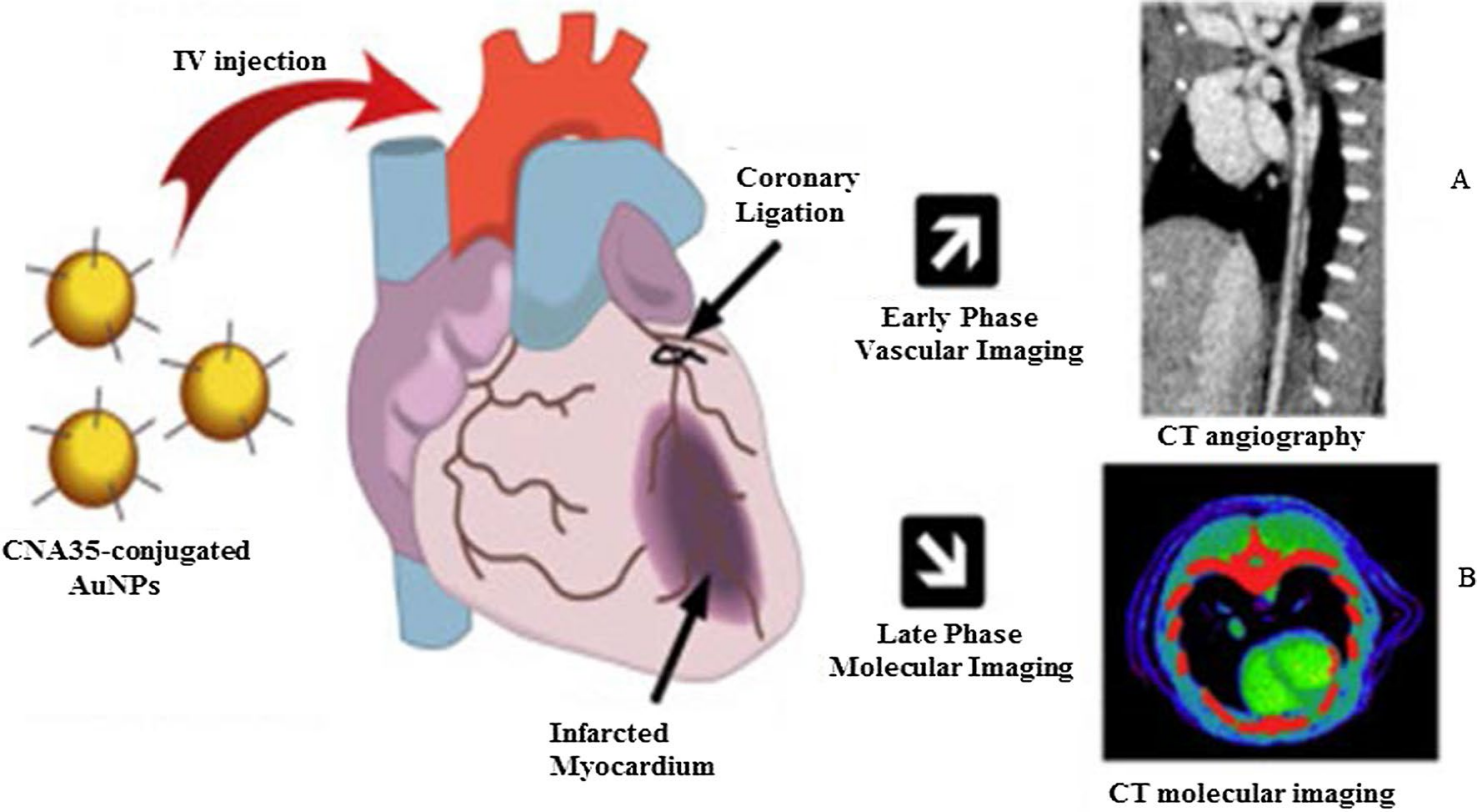
In vivo CT imaging using AuNPs as CT contrast agents. Mannan-capped AuNPs and their CT imaging of the lymph node (**A**), and CNA35-conjugated AuNPs CT imaging of myocardial scar burden (**B**). Reproduced with permission [79]. Copyright 2018, Elsevier

Figure 5 shows an in vivo CT vascular imaging of coronary arteries using AuNPs that were conjugated to collagen-binding adhesion protein 35 (CNA35) for targeting collagen I in myocardial infarction in rodents. The AuNP signal was still detected in the blood 6 h after intravenous (i.v) administration, which was significantly higher than the half-life (5–10 min) of iodine-based agents [79]. These effects were replicated by using green-synthesized mannan-capped AuNPs, which showed receptor-mediated uptake and non-toxicity in mannose expressing (DC 2.4 and RAW 264.7) cells. The mannan-capped AuNPs selectively targeted the popliteal lymph nodes in vivo after injection into the hind leg of the mice [38]. The AuNP-based CT imaging can provide significant information for diagnosis of various diseases not limited to coronary artery and cancers [76, 77, 79–81]. The use of AuNPs as contrast agents has shown potential in other imaging systems such as photoacoustic, nuclear imaging, ultrasound and magnetic resonance imaging. These systems are extensively reviewed elsewhere [82, 83].

#### AuNPs in Fluorescent-Based Detection Systems

AuNPs are used in fluorescent-based detection systems as either fluorescent agents or fluorescent quenchers. At sizes ≤ 5 nm, AuNPs display properties of quantum dots (QDs) and can be used in their place. The Au_55_(PPh_3_)_12_Cl_6_ nanoclusters introduced in 1981 are probably the most intensively studied owing to their quantum size behavior [7]. Since then, various quantum-sized AuNPs (AuNPsQ) such as Au_25_(SR)_18_, Au_38_(SR)_24_ and Au_144_(SR)_60_ [84] have been studied mostly in electrochemical sensing as they are excellent electronic conductors and redox mediators [85].

AuNPsQ film electrodes were used in the fabrication of an ultrasensitive electrochemical immunosensor for the detection of prostate-specific antigen (PSA). The immunosensor had a sensitivity of 31.5 μA mL/ng and a detection limit of 0.5 pg/mL for PSA in 10 μL of undiluted human serum. The immunoassay performed eightfold better than a previously reported carbon nanotube forest immunosensor containing multiple moieties, at the biomarker concentration that was lower than the levels associated with the presence of cancer. As such, it can be used to measure the test biomarker in both normal and diseased states. The performance of the immunosensor was comparable to the reference ELISA method [86]. AuNPsQ was also incorporated into porously structured CaCO_3_ spheres to form a fluorescent CaCO_3_/AuNPsQ hybrid for the detection of neuron-specific enolase, a diagnostic and prognostic biomarker for traumatic brain injury and lung cancer. The sensor had a detection limit of 2.0 pg mL^−1^ [87]. Until now, several AuNP-based fluorescent detection systems have been reported for the detection of analytes associated with Hepatitis B [73, 88], Influenza A [89], cancer [90] and heart injury [91].

AuNPs are also excellent FRET-based quenchers [92]. Their unique optical properties (stable signal intensity and photobleaching resistance), size and ability to be modified have made them attractive probes in fluorescence sensing platforms [93, 94]. Larger AuNPs (≥ 10–100 nm) have low quantum yields that are not suitable for direct fluorescent sensing; however, their ability to quench fluorescent dyes under relatively high excitation energy state has made them effective photoluminescence quenchers [94]. In principle, fluorescence nanoprobes are composed of a donor fluorophore (dye or QDs) and an acceptor AuNPs, and when brought into close proximity, the fluorescence of the selected fluorophore is quenched by the AuNPs [94, 95]. In the absence of a target as indicated by a lack of a fluorescent signal, the nucleic acid probe hybridizes and forms a looped structure that brings the fluorophore and a quencher at its opposite ends into close proximity; while binding of the analyte to the nucleic acid probe displaces the fluorophore from the AuNPs resulting in a fluorescent signal [24, 94, 96]. Taking advantage of the above-mentioned properties, AuNPs were incorporated in molecular beacons for in vitro (gold nanospheres, AuNSs) and in vivo (gold nanorods, AuNRs) detection of the matriptase expression on tumor cells. The two molecular beacons were composed of a matriptase cleavage site as a linker between the AuNPs and the fluorophores. The AuNS–molecular beacon was constructed with the fluorescein isothiocyanate (FITC), and the AuNR–molecular beacon had a NIR fluorescent dye (mercaptopropionic acid, MPA). In the absence of the target, the AuNSs and AuNRs, respectively, blocked the FITC and MPA fluorescence. Cleavage of either FITC or MPA from the AuNP–molecular beacons in the presence of matriptase exhibited a quantifiable fluorescence signal. The fluorescent signal of the MPA–AuNR–beacon in the nude mice bearing HT-29 tumors lasted for 14 h in the tumor site, while the signal gradually disappeared from the non-tumor site over time [97].

The AuNPs were reported to have comparable or higher fluorescence quenching efficiency than organic quenchers such as 4-((4′-(dimethyl-amino)phenyl)azo)benzoic acid (DABCYL) [94, 98] and Black Hole Quencher-2 [99]. The fluorescence quenching efficiency of 1.4 nm AuNPs was compatible with the four commonly used organic fluorophores (FITC, rhodamine, texas red and Cy5). The fluorescence quenching efficiency of the AuNPs was similar to that of DABCYL, and unlike DABCYL, the AuNPs showed consistency in both low and high salt buffers [98]. In a competitive hybridization assay, 10 nm AuNPs showed superior (> 80%) fluorescence quenching efficiency for Cy3 dye than the commercial Black Hole Quencher-2 (~ 50%). The assay had a limit of detection of 3.8 pM and a detection range coverage from 3.8 pM to 10 nM for miRNA-205 in human serum, and it was able to discriminate between miRNAs with variations in their nucleotide sequence [99]. The competitive sensor arrays were not only sensitive [96, 99] but were able to differentiate between normal and diseased cells, as well as benign and metastatic cancers [96].

#### AuNP-Based Bio-barcoding Assay

AuNP-based bio-barcoding assay (BCA) technology has become one of the highly specific and ultrasensitive methods for detection of target proteins and nucleic acids up to 5 orders of magnitude than the conventional assays [100]. The assay relies on magnetic microparticle probes, which are functionalized with antibodies that bind to a specific target, and AuNP probes encoded with DNA that recognizes the specific protein target and antibodies. Upon interaction with the target DNA, a sandwich complex between the magnetic microparticle and AuNPs probes is formed. The sandwich is then separated by the magnet followed by thermal dehybridization to release the free bar-code DNA, enabling detection and quantification of the target [101, 102].

The AuNP-based BCA assay was able to detect HIV-1 p24 antigen at levels that was 100–150-fold higher than the conventional ELISA [103]. The detection limit of PSA using these systems was 330 fg/mL [104]. The versatility of AuNPs for the development of a BCA-based platform was further demonstrated by measuring the concentration of amyloid-beta-derived diffusible ligands (ADDLs), a potential Alzheimer's disease (AD) marker found in the cerebrospinal fluid (CSF). ADDL concentrations were consistently higher in the CSF taken from the subjects diagnosed with AD than in non-demented age-matched controls [105]. These results indicate that the universal labeling technology can be improved through the use of AuNPs to provide a rapid and sensitive testing platform for laboratory research and clinical diagnosis.

### AuNP-Based Therapies

Metal-based drugs are not new to medicine; in fact, they are inspired by the existing metallic drugs used in clinical treatment of various diseases [9, 106–109]. The widely studied and clinically used metal-based drugs were derived from platinum (e.g., cisplatin, carboplatin, tetraplatin for treatment of advanced cancers), bismuth (for the treatment of infectious and gastrointestinal diseases), gold (for the treatment of arthritis) and gallium (for the treatment of cancer-related hypercalcemia) [108, 109]. The approval of cisplatin in 1978 by the FDA for the clinical treatment of cancer [107] further inspired research on other metals (such as palladium, ruthenium, rhodium) [32, 106, 110].

Owing to the bioactivities, which included anti-rheumatic, antibacterial and anticancer effects, and the biocompatibility of bulk gold [8, 9, 46, 111], AuNPs are extensively investigated for the treatment of several diseases. AuNPs displayed unique and novel properties that are superior to its bulk counterpart. AuNPs are highly stable and have a distinct SPR, which guides their application in medicine [112], as drug delivery and therapeutic agents. AuNPs have a lot of advantages over the conventional therapy; they have a longer shelf-life and can circulate long enough in the system to reach their targets [25] with [11, 49, 113] or without targeting molecules [14, 15, 24, 25, 114]. AuNPs can provide localized and selective therapeutic effects; some of the areas in which AuNPs were used in therapy are described below.

#### Therapeutic Effects of Untargeted AuNPs

The as-synthesized (i.e., unmodified or uncapped) AuNPs have been shown to have diverse therapeutic effects against a number of infectious [115, 116], metabolic and chronic diseases [3, 29, 50, 51]. Their antioxidant, anticancer, anti-angiogenic [3, 32], anti-inflammatory [3, 51] and weight loss [29, 50, 112] effects are beneficial for diseases such as cancer, rheumatoid arthritis, macular degeneration and obesity [5, 25, 113, 117]. The above-mentioned diseases are characterized by a leaky vasculature and highly vascularized blood vessels [5, 113], which provides the NPs an easy passage into the diseased tissues and increase the susceptibility of cells to their effects. Through the EPR effect, uncapped AuNPs can passively accumulate in the vasculature of diseased cells or tissues. Hence, AuNPs have been specifically designed to have anti-angiogenic effects in diseases where angiogenesis (the growth and extension of blood vessels from pre-existing blood vessels) spins out of control like cancer, rheumatoid arthritis, macular degeneration and obesity [5, 25, 113, 117]. Targeting and destroying the defective blood vessels prevent oxygen and nutrients from reaching the diseased cells, which results in their death. The pores in the blood vessels at the diseased site (especially in cancer and obesity) are 200–400 nm and can allow materials in this size range to pass from the vasculature into the diseased tissues and cells [14, 15, 25, 114].

The cellular uptake, localization, biodistribution, circulation and pharmacokinetics of the uncapped AuNPs rely strongly on size and shape [49]. Although these effects are applicable to all AuNPs, the biological effects of citrate-capped AuNPs (cAuNPs) are extensively studied and reviewed. Spherical cAuNPs demonstrated selective in vitro anticancer activity that was size and concentration dependent on murine and human cell lines [3, 51]. Different sizes (10, 20 and 30 nm) of cAuNPs showed differential effects in human cervical carcinoma (HeLa), murine fibroblasts (NIH3T3) and murine melanoma (B16F10) cells. The 20 and 30 nm cAuNPs showed a significant cell death in HeLa cells starting at the lowest concentration of 2.2 µg/mL, while the 10-nm NPs was toxic at concentrations ≥ 8.75 µg/mL. The activity of these NPs was negligible in the noncancerous NIH3T3 cells, especially the 10 and 20 nm. The 20 nm reduced viability by ≤ 5% at the highest concentration (35 µg/mL), and ~ 20% for the 10 and 30 nm. The IC_50_ values for 10, 20 and 30 nm cAuNPs in the Hela cells were 35, 2.2 and 4.4 μg/mL, respectively, while the IC_50_ values for noncancerous cells were higher than 35 µg/mL [3]. Using a concentration range of 0.002–2 nM, 13 nm cAuNPs induced apoptosis in rabbit articular chondrocytes and no effects were observed for 3 and 45 nm cAuNPs under the same conditions. The 13 nm cAuNPs induced mitochondrial damage and increased reactive oxygen species (ROS); these actions could not be blocked by pre-treatment with a ROS scavenger, the N-acetyl cysteine [51]. Size-dependent effects were also observed in vivo after injecting cAuNPs of various sizes (3, 5, 8, 12, 17, 37, 50 and 100 nm) into mice (8 mg/kg/week) for 4 weeks. The 8, 17, 12 and 37 nm were lethal to the mice and resulted in tissue damage and death after 14 days of treatment; the other sizes were not toxic and the mice survived the experimentation period. On the contrary, the same-size AuNPs at a concentrations up to 0.4 mM were not toxic to HeLa cells after 24 h exposure [118].

The cAuNPs can interact and accumulate nonspecifically within various tissues and organs in the body, especially in the reticuloendothelial system (RES) organs (blood, liver, spleen, lungs) [55, 119]. This was evident in high-fat (HF) diet-induced obese Wistar rats [55] and Sprague–Dawley rats [119] following acute (1 dose for 24 h) [55] and chronic (1 dose; 0.9, 9 and 90 µg/week over 7 week period) [119] exposure to 14 nm cAuNPs, respectively. Majority of the i.v injected cAuNPs were detected in the liver, spleen, pancreas, lungs, kidneys [55, 119] including the skeleton and carcass of the rats [119]. Chen et al. observed that after intraperitoneal (i.p) injection of a single dose (7.85 µg/g bodyweight) of 21 nm cAuNPs in lean C57BL/6 mice, they accumulated in the abdominal fat tissues and liver after 24–72 h [29], as well as the spleen, kidney, brain and heart in the HF-induced obese mice that were injected with the same dose daily for 9 weeks [50]. The cAuNPs reduced the abdominal WATs (retroperitoneal and mesenteric) mass and blood glucose levels 72 h post-injection [29]. In the diet-induced obese mice, the 21 nm cAuNPs demonstrated anti-inflammatory and anti-obesity effects [50]. They also improved glucose tolerance, enhanced the expression of inflammatory and metabolic markers in the retroperitoneal WATs and liver [50]. Both the 14 and 21 nm cAuNPs showed no sign of toxicity or changes in the markers associated with kidney and liver damage [29, 55, 119].

**Fig. 6:**
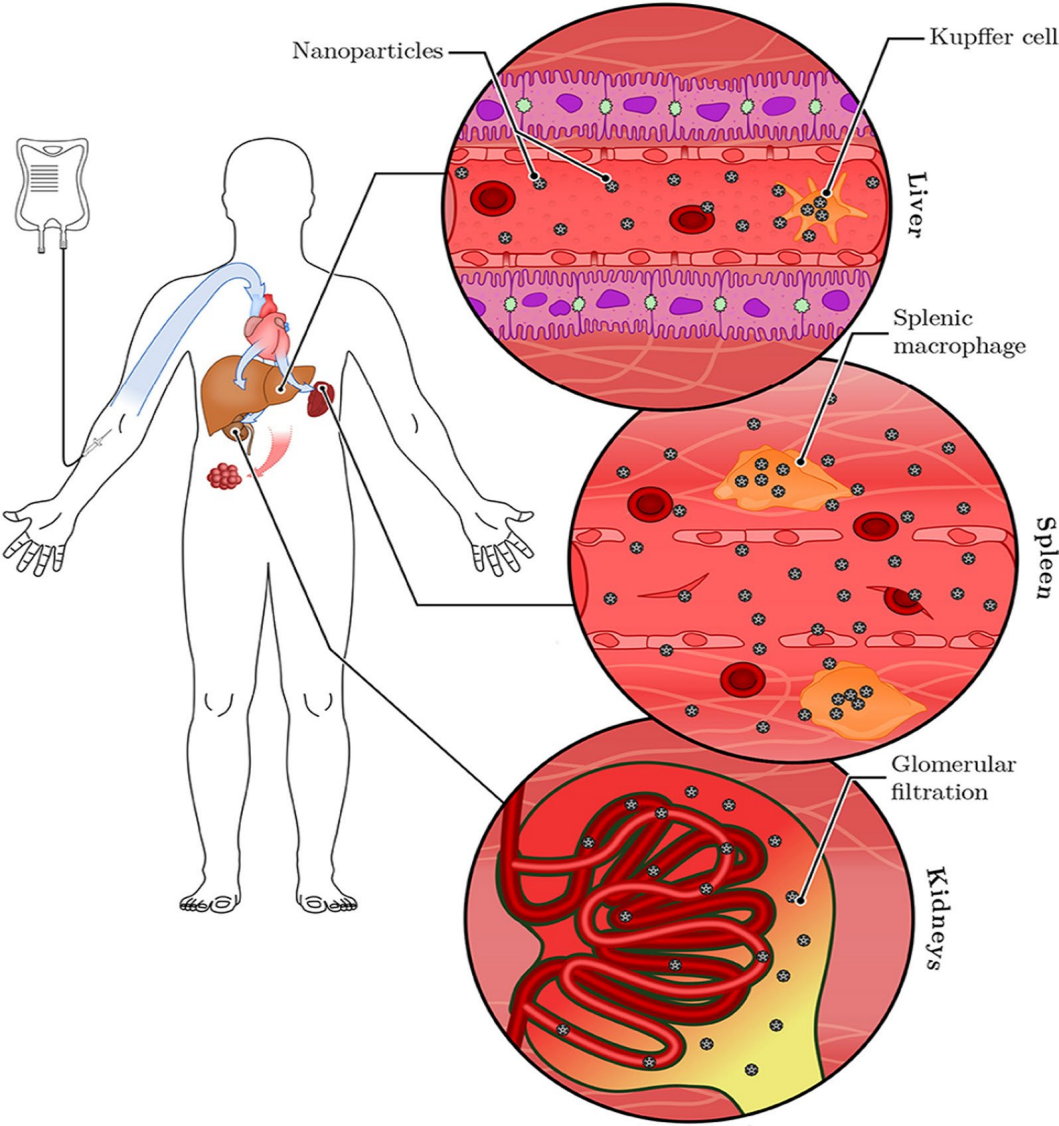
RES-based clearance of systemic administered AuNPs depends on their size. Large AuNPs accumulate in the liver, while smaller AuNPs are likely to end up in the spleen or be excreted in the urine via glomerular filtration. The AuNPs that escape the TRMs could accumulate in the diseased tissues. Reproduced with permission [114]. Copyright 2019, Frontiers in Bioengineering and Biotechnology

Similar findings were reported for plant-mediated AuNPs, without targeting molecules they can access, ablate tumors [40, 120] and obese WATs [121] in rodents. Differential uptake, distribution and activity of biogenic AuNPs also vary depending on the size and shape of the NPs. While certain sizes can pass through the vascular network and be retained at the site of the disease; others can be easily filtered out of the system through the RES organs and the mononuclear phagocytic system as shown in Fig. 6 [15, 114]. NPs can be removed by tissue-resident macrophages (TRMs) before they reach the disease cells. Those that escape the TRMs and do not reach the disease site, especially smaller NPs (≤ 5 nm), are excreted through glomerular filtration in the kidney [25, 114]. Pre-treatment with clodronate liposomes depleted the TRMs in the liver and spleen before exposure to 50, 100 and 200 nm AuNPs. This reduced uptake of the AuNPs by the liver, increased their half-life in the blood as well as their accumulation at the tumor site [122]. However, TRMs are not the only obstacle that the AuNPs that rely on EPR effect for uptake must overcome. EPR effect alone can only ascertain ≤ 1% AuNP uptake [15, 114], and depletion of the TRMs prior to treatment resulted in just ≤ 2% of NPs reaching the target [122]. The success of non-targeted AuNPs depends on their ability to reach and accumulate in the diseased tissues, of which passive targeting through the EPR effect might not be efficient. The NPs also need to circulate longer, escape early clearance, and most importantly show reduced bystander effects [25, 123]. These qualities can increase bioavailability and ensure selectivity and efficacy of the AuNPs. These can further be improved by changing the surface chemistry of the AuNPs as discussed below [15, 124].

#### Therapeutic Effects of Surface-Functionalized AuNPs

The common strategy in AuNP-based therapeutics involves modifying the AuNP surface with therapeutic agents [3, 124–126]. The therapeutic agents can be drugs already used for the treatment of a particular disease or biomolecules with known inhibitory effects on cell signaling. In some instances, the therapeutic AuNPs have also been designed to have molecules that facilitate active targeting of the AuNPs toward specific cells and tissues. The molecules can easily adsorb on the AuNP surface by thiolation, chemical modification using chemistries such as 1-ethyl-3-(3-dimethylaminopropyl) carbodiimide (EDC), streptavidin/biotin binding [3, 124–127] and ionic interactions based on opposite charges between the NP surface and the biomolecules [124–126]. Functionalization of the AuNP surface influences their physicochemical properties and can affect their safety, biocompatibility and mobility. To ensure that the cargo carried by the AuNPs is delivered to the intended site, consideration should thus be given to both the physical and chemical properties of the AuNPs [124–126]. It is especially the size, shape, charge and the capping agents of the AuNPs that play an important role in the functionality of the AuNP conjugates [124] and can completely alter the pharmacokinetics of the AuNP-based therapeutics.

Functionalization allows for the development of customized nanosystems to reduce undesirable bystander effects often associated with traditional medicine. Functionalization of AuNPs can also prevent nonspecific adsorption of proteins onto the AuNP surface which can result in the formation a protein corona, resulting in the early clearance of the AuNPs through opsonization by the phagocytic cells [49, 123]. The surface charge of NPs can have a major influence on the behavior of NPs within biological environments. AuNPs with a neutral surface charge are unreactive and have a higher rate of escaping opsonization than charged AuNPs. Hydrophilic NPs will also behave differently to those with hydrophobic surfaces [49, 123]. PEG is one of the polymers most often used to mask AuNPs from phagocytic cells and has been shown to stabilize and enhance the biocompatibility of the AuNPs in numerous in vivo studies [49, 55, 123]. Pegylation improved the biocompatibility of 8.2 nm AuNPs by preventing neutrally and negatively charged AuNPs to bind to cell membranes or localize to any cellular components in African green monkey kidney (COS-1) cells [49]. And when the pegylated AuNPs were functionalized with a polyarginine cell penetrating moiety, the AuNPs were visualized on the cell membrane and inside the COS-1 cells [49]. Cell-penetrating peptides such as nuclear localization signal from SV40 virus, Tat from HIV and polyarginine peptides have been explored in translocation of AuNPs inside all cell type, normal or diseased. However, high specificity is required for clinical applications and can be achieved by taking advantage of the physiological differences between malignant and normal cells. This has been achieved by functionalizing the AuNPs with targeting molecules that recognize cell-specific receptors that are exclusively or overexpressed on the surface of target cells. This way, the AuNPs can be directed and delivered only to cells that express the target receptor. Therefore, conjugation of targeting moieties to the AuNPs (active targeting) will provide more selectivity, reduced bystander toxicity and enhanced efficacy since the AuNPs will be confined only to malignant tissues that express the target receptors [49, 55, 57, 113, 126, 127].

**Fig. 7:**
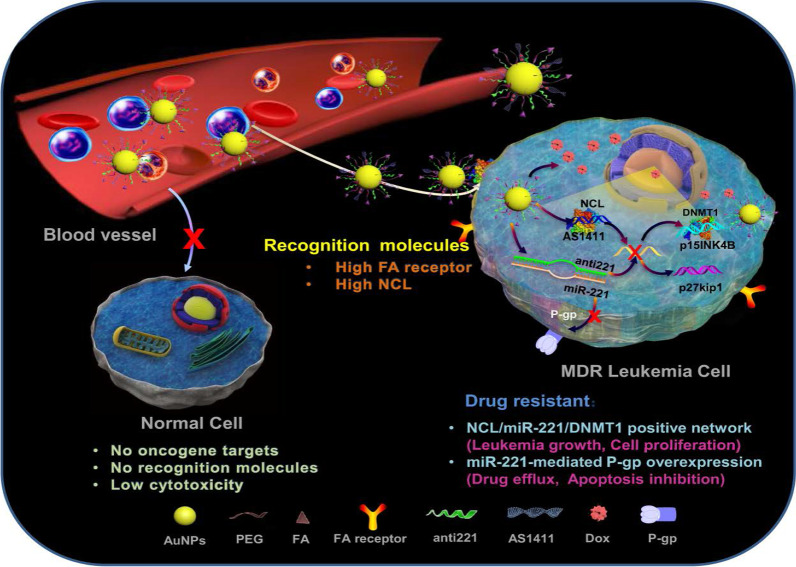
Multifunctional AuNPs in the treatment of multidrug-resistant (MDR) leukemia cells by increasing the sensitivity of the cells to Dox. Reproduced with permission [11]. Copyright 2019. Springer Nature. Folate (FA) receptor

A good example to demonstrate the versatility of AuNPs is shown in Fig. 7, where four different molecules were conjugated onto the AuNPs to target two independent markers and mechanisms [11]. The multifunctional AuNPs were used for the treatment of leukemia (K562DR) cells that are resistant to doxorubicin (Dox). The 40 nm AuNPs were modified with two targeting moieties (folate and AS1411 aptamer) and two therapeutic agents (Dox and anti-miRNA molecules/anti-221). Folate molecule and AS1411 aptamer, respectively, recognize the folate and NCL receptors that are overexpressed on the cell surface and through receptor-mediated endocytosis will traffic the AuNP-conjugate into the cells. The AS1411 aptamer had dual functions, by also targeting the NCL receptor that is expressed inside the cells. After the AuNP-conjugate has been shuttled into the cells, the cargo (AS1411 aptamer, anti-221 and Dox) is off-loaded which independently act on three mechanisms that will synergistically bring about the demise of the cells. AS1411 aptamer together with anti-221 prevented leukemogenesis by suppressing the endogenous NCL and miR-221 function in the NCL/miR-221 pathway, thereby sensitizing the cells to the effects of Dox [11].

Interestingly, similar dual targeting and treatment effects were achieved with green synthesized AuNPs without any additional molecules. With natural products acting as reducing agents, the biogenic AuNPs might also be more biocompatible than the chemically synthesized NPs [12, 40, 41, 43, 120]. MGF-AuNPs selectively targeted the laminin receptors in prostate (PC-3) and triple-negative breast cancer (MDA-MB-231) cells, and their xenografts in severe combined immunodeficiency (SCID) mice bearing these tumors [12, 40, 120]. In the normal SCID mice, the majority (85% at 30 min increasing to 95% after 24 h) of the i.v-injected MGF-AuNPs accumulated in the liver. Less than 10% were detected in the blood (2.7%), spleen (5%), lungs (0.6%), stomach, intestines and kidneys. When intra-tumorally injected in SCID mice-bearing prostate tumors, only 11% of the MGF-AuNPs were detected in the liver 24 h post-injection, while ~ 80% was in the tumor. Negligible amounts were found in the stomach, carcass and the small intestines. Some of the AuNPs were excreted through the renal and hepatic pathways in the urine and feces after 24 h [40, 120]. Nano Swarna Bhasma, a mixture consisting of AuNPs synthesized from mango peel extracts and phytochemicals from mango, turmeric, gooseberry and gum arabic, showed reduced toxicity toward normal endothelial cells after 48 h compared to the MDA-MB-231 cells [12].

Several studies have demonstrated that AuNPs have potential for clinical application. In combination with conventional drugs, it can be used to sensitize diseased cells to the drug effects [12, 128] and also prevent or reduce drug-related bystander effects [12]. AuNPs improved the pharmacokinetics of chemotherapeutic drugs, such as Dox [43, 129] and 5-fluorouracil (5-FU) [128]. Great improvements were mostly seen in the permeability and retention of drugs in the diseased cells, resulting in enhanced efficacy [130]. Dox-loaded AuNPs, which were non-toxic toward normal mouse fibroblast (L929) cells, also demonstrated selective toxicity toward fibrosarcoma tumors in mice [129]. 5-FU conjugated to the cAuNPs had better activity than 5-FU on its own in colorectal cancer cells [128]. AuNP co-treatment with chemotherapeutic drugs was highly efficient in improving the efficacy of chemotherapeutic drugs [12, 43, 128, 129, 131]. Orally ingested Nano Swarna Bhasma in combination with Dox and Cyclophosphamide reduced tumor volumes in SCID mice-bearing breast tumor cells and also showed acceptable safety profile and reduced bystander effects of the chemotherapeutic drugs in stage IIIA/B metastatic breast cancer patients [12]. Active targeting alone can ensure that the AuNPs are directly delivered into the desired targets, achieving a balance between efficacy and toxicity while minimizing damage to healthy tissues [14, 15, 49]. Controlled drug release is also among the many advantages offered by the AuNP-based systems and is crucial as it allows for localized and selective toxicity [49]. The AuNPs can be designed in such a way that their conjugates respond to internal (glutathione displacement, enzyme cleavable linkers, pH) or external (light, heat) stimuli to function [24, 25, 34, 128].

#### AuNPs as Transfection Agents in Gene Therapy

The use of AuNPs in gene therapy has shown promising outcomes by facilitating the delivery of genetic material to cells to silence or enhance expression of specific genes [24, 32, 132]. Thus, AuNPs can be used as transfection reagents in gene therapy for the treatment of cancer and other genetic disorders. AuNP conjugates have demonstrated higher transfection efficiency than experimental viral and non-viral gene-delivery vectors including polycationic reagents that has been approved for clinical use [24].

AuNPs are highly conductive and well suited for use as microelectrodes during electroporation for intracellular delivery of biomolecules for disease treatment. AuNPs significantly enhanced the performance of electroporation systems and have been used successfully for the delivery of DNA into hard-to-transfect cells such as the K562 cells [133]. To prevent cell loss which is often associated with electroporation, targeting moieties can be conjugated to the AuNPs to facilitate cellular uptake of AuNP conjugates through receptor-mediated mechanisms [133]. The use of AuNPs to transfect cells with oligonucleotide molecules also has the added advantage of increasing the half-life of these biomolecules and their efficacy [24, 32].

Untargeted AuNP conjugates are passively transported into cells and rely on the surface charge and AuNP shape for efficient transfection [24, 36, 134, 135]. The charge of the biomolecules that are conjugated onto AuNP surface plays a crucial role in their transfection efficiency; for instance, AuNPs functionalized with cationic molecules produce higher transfection efficiency than AuNPs functionalized with anionic molecules. Positively charged amino acids (lysine) can be attached on the NP surface to increase the rate of transfection. AuNSs [24] and AuNRs [36, 134, 135] are commonly used for transfections, and relative to the conventional transfection reagents (X-tremeGENE and siPORT), they inhibited the expression of target gene by > 70% in vitro [134] and in vivo [135]. In these studies, transfection efficiency was quantified based on target expression using RT-PCR and immunostaining [134, 135]. As transfection reagents, AuNPs provide long-lasting effects, localized gene delivery and higher efficacy [36, 134, 135]. Other types of nanomaterials (e.g., polymeric, liposomes, ceramic and carbon nanotubes) had received more attention for use in gene therapy than AuNPs. Six clinical trials using either polymeric or lipid-based nanomaterials for delivery of siRNA in solid tumors have been completed [36, 134, 136]. All of which suffer from low loading efficiency, low stability, and insufficient payload release [36, 136]. On the other hand, transfection systems based on AuNPs make use of easy chemistry that ensures efficient loading capacity and formation of stable complexes [36, 135]. Their safety can be controlled by manipulating their shape, size distribution and surface composition [36].

#### Antimicrobial Effects of AuNPs

MDR microbes are a major health concern and a leading cause of mortality, worldwide [21, 137–141]. These microorganisms have become resistant to conventional antimicrobial agents, due to over-prescription and misuse of these drugs [142]. No new antibiotics have been produced in over 40 years, mainly because the big pharmaceutical companies have retreated from their antibiotic research programs due to the lack of incentives [143]. As such, new and effective antimicrobial agents are urgently required to combat what could be the next pandemic, the antimicrobial resistance, and avoid surge in drug-resistant infections.

**Fig. 8:**
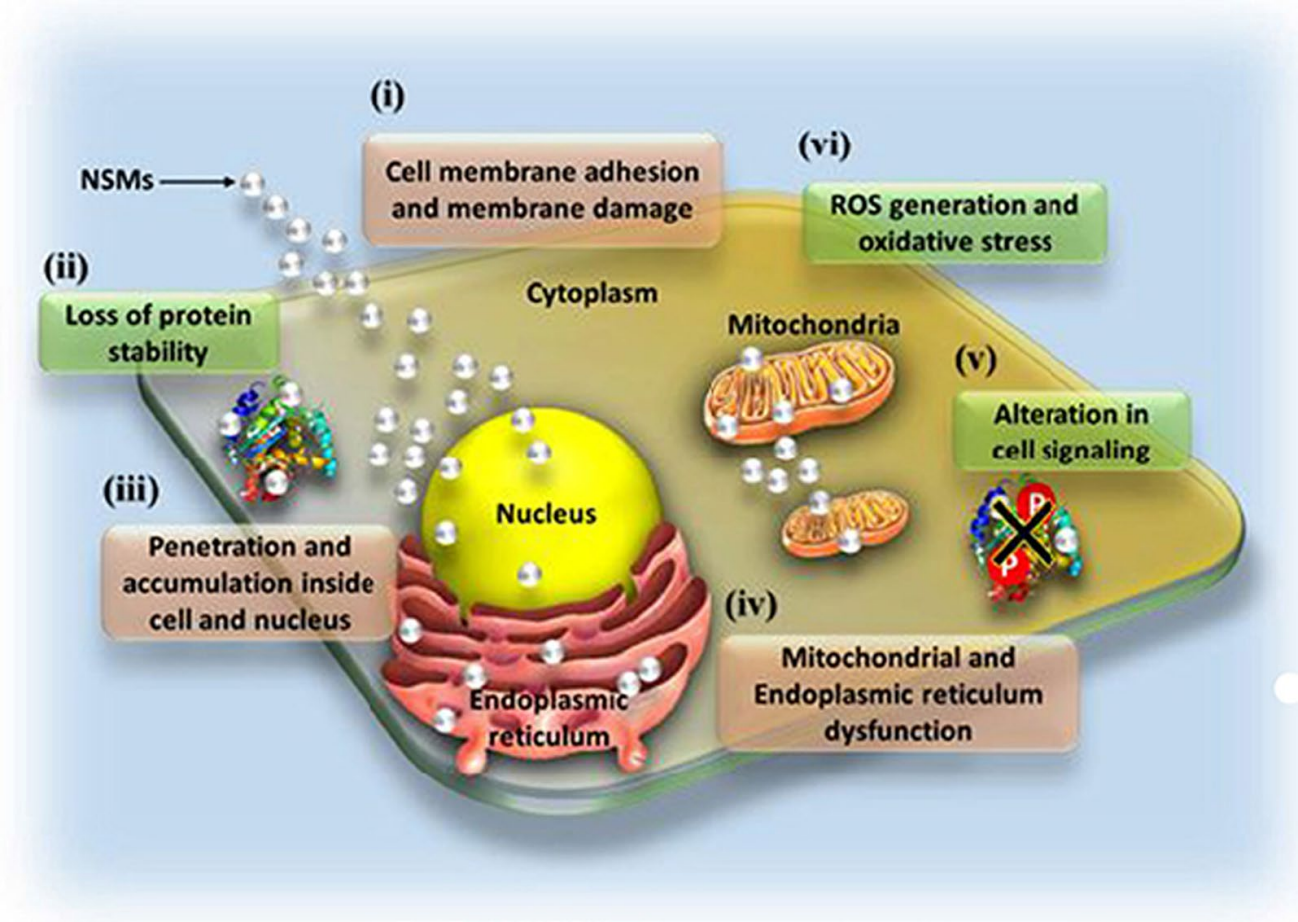
Antimicrobial mode of actions of the NSMs. Various NSMs can induce cell death by altering various biological functions, X represents alteration of cell signaling by de-phosphorylation of tyrosine residues in proteins as one of the mechanisms. Reproduced with permission [141]. Copyright 2018, Frontiers in Microbiology

AuNPs are among the new generation of antimicrobial agents under review. They have shown broad antimicrobial (bactericidal, fungicidal and virucidal) effects against a number of pathogenic and MDR microorganisms and thus have potential to overcome microbial drug resistance [21, 142, 144]. Their antimicrobial effects are dependent on their physicochemical properties, especially their size, surface composition, charge and shape [21, 144]. Due to their small size, AuNPs can easily pass through the bacterial cell membrane, disrupt their physiological functions and induce cell death [35]. The exact antimicrobial mechanisms of AuNPs are not yet fully elucidated; despite this, some of the reported modes of actions that results from the interaction of various nanostructured materials (NSMs) with the bacterial cells are illustrated in Fig. 8. The highlighted mechanisms are also implicated in antimicrobial activity of AuNPs, they include induction of microbial death through membrane damage, generation of ROS and oxidative stress, organelle dysfunction, and alteration of gene expression and cell signaling [141].

AuNPs have multiple roles to play toward the development of antimicrobial agents, aside from being antimicrobial agents by themselves; they can serve as drug sensitizers and drug delivery vehicles [35, 58, 132, 145]. These features are applicable to both the chemical and green synthesized AuNPs, which have been reported to have antimicrobial effects against a number of human [21, 145–147] and waterborne [148] pathogenic strains. Generally, the test bacteria had shown low susceptibility toward the chemically synthesized AuNPs, i.e., the cAuNPs [21, 146, 147] and the NaBH_4_-reduced AuNPs [149]. This was due to the repulsive forces between the negative charges on the AuNP surfaces and bacterial cells, thus preventing the interaction between AuNPs and the bacteria [21]. The activity of chemically synthesized AuNPs is based on their size, shape, concentration and exposure time. As an example, one study reported that NaBH_4_-reduced AuNPs had no activity against *Staphylococcus aureus* (*S. aureus*) and *Escherichia coli* (*E. coli*) at 500 µg/mL for the duration of 6 h [149]. In contrast, another study showed a significant dose (1.35, 2.03 and 2.7 μg/mL) and size (6–34 nm vs 20–40 nm) dependent antibacterial effects of the NaBH_4_-reduced AuNPs on *Klebsiella pneumonia*, *E. coli*, *S. aureus* and *Bacillus subtilis* [145].

The AuNPs are either used alone or in combination with other antimicrobial agents to treat microbial infections [35, 58, 132, 145]. When used in combination with other antimicrobial agents, the AuNP conjugates resulted in synergistic antimicrobial effects that surpassed the individual effects of the AuNPs and drugs [21, 35, 58, 132, 150]. These drugs were conjugated onto the AuNPs by either chemical methods [4, 151] or the drugs were used as reducing and capping agents [21, 149]. By so doing, the AuNPs improved drug delivery, uptake, sensitivity and efficacy. Some of the FDA-approved antibiotics and non-antibiotic drugs that were loaded onto the AuNPs are shown in Table 1 [4, 21, 149, 152]. Ciprofloxacin [152], cefaclor [149], lincomycin [4], kanamycin [21], vancomycin, ampicillin [151] and rifampicin [32] are among the antibiotics loaded on the AuNPs and demonstrated the versatility of AuNPs. These strategies were successful with various sizes and shapes of AuNPs, including gold silica nanoshells [152], AuNP-assembled rosette nanotubes [151] and AuNPs encapsulated in multi-block copolymers [153]. For instance, cefaclor-reduced AuNSs inhibited the growth of *S. aureus* and *E. coli* within 2–6 h depending on the concentration (10–50 µg/mL), while complete bacterial growth inhibition by the drug alone was only observed at 50 µg/mL after 6 h. The minimum inhibitory concentration (MIC) of the treatments was 10 µg/mL and 50 µg/mL for cefaclor-AuNPs and cefaclor, respectively [149].

**Table 1:** Antimicrobial activities of AuNP loaded with some of the FDA-approved drugs

Drug	Drug type	Core sizes (nm)	Shape	Test microorganisms	Ref
Ciprofloxacin	Antibiotic	15	Gold silica nanoshells	*E. coli* DH5α *Lactococcus lactis*	[152]
Cefaclor	Antibiotic	23–52	Spheres	*S. aureus* *E. coli*	[149]
Lincomycin	Antibiotic			*S. aureus* *S. pyogenes*	[4]
Vancomycin	Antibiotic			Vancomycin-resistant strains *(VRE)*	[150]
Ampicilin	Antibiotic	1.43	Spheres assembled into rosette nanotube	*S. aureus* MRSA	[151]
Rifampicin	Antibiotic	28	Spheres	N/T	[153]
Kanamycin	Antibiotic	20	Spheres	*Streptococcus bovis (S. bovis)* *Staphylococcus epidermidis (S. epidermidis)* *Enterobacter aerogenes (E. aerogenes)* *Pseudomonas aeruginosa* (*P. aeruginosa*) *Yersinia pestis (Y. pestis)*	[21]
4,6-diamino-2-pyrimidinethiol (DAPT)	Inactive alone	5 / 16.57	Spheres	*S. aureus* *E. coli* *P. aeruginosa* *E. coli* infected mice	[13, 112]
5FU	Anti-leukemic drug	18	Spheres	*Micrococcus luteus* *S. aureus* *P. aeruginosa* *E. coli* *Aspergillus fumigatus* *Aspergillus niger*	[58]
Metformin	Anti-hyperglycemic drug	3.1		*S. aureus* *E. coli* *P. aeruginosa*	[13]

*N*/*T* not tested

AuNPs have presented properties that make them ideal candidates as alternative antimicrobial agents; the most important being their broad antimicrobial activity [21, 35, 58, 132, 150]. Owing to their biocompatibility and easily modifiable surface, microorganisms are less prone to developing resistance toward AuNPs [21]. For example, the kanamycin (Kan)-resistant bacteria (*S*. *bovis*, *S*. *epidermidis*, *E*. *aerogenes*, *P*. *aeruginosa* and *Y*. *pestis*) showed increased susceptibility toward Kan-reduced AuNPs. The MIC values for Kan-AuNPs on the test bacteria were significantly reduced to < 10 µg/mL when compared to the MIC values for Kan alone at 50–512 µg/mL. This shows that AuNPs can restore the potency of antibiotics toward the drug-resistant strains by facilitating the uptake and delivery of the antimicrobial agents [21]. AuNPs can enhance drug-loading capacity and control the rate at which the drugs are released. AuNP hybrids with the multi-block copolymers increased the loading capacity of rifampicin and the drug’s half-life to 240 h. By sustaining the drug in the system for that long, ensured slow release of rifampicin from AuNPs at the target sites after oral administration of the AuNP conjugates to rats for 15 days. The drug on the surface was released within 24 h followed by the drug trapped in the polymer matrix after 100 h. And lastly, the drug entrapped between the AuNPs and the polymer matrix took over 240 h to be released in the interstitial space [153].

The AuNP hybrids also allow for the conjugation of multiple molecules with independent but synergistic functions. This was demonstrated by co-functionalization of the AuNPs with antimicrobial peptide (LL37) and the pcDNA that encode for pro-angiogenic factor (vascular endothelial growth factor, VEGF) and used in the treatment of MRSA-infected diabetic wounds in mice [132]. The AuNPs served dual functions, as a vehicle for the biomolecules, and also as transfection agent for the pcDNA. After topical application of the AuNP conjugates on the wound, the LL37 reduced MRSA colonies, while the pcDNA promoted wound healing by inducing angiogenesis through the expression of VEGF [132].

AuNPs have been shown to confer activity and repurpose some non-antibiotic drugs toward antimicrobial activity. The examples of repurposed drugs, which were used for the treatment of diseases other than bacterial infections, include 5FU [58], metformin [147] and 4,6-diamino-2-pyrimidinethiol (DAPT) [13, 112]. AuNPs as drug carriers are able to transport the drugs into the cells and allow direct contact with cellular organelles that resulted in their death [58, 147]. 5FU is an anti-leukemic drug, when attached to AuNPs was shown to kill some bacterial (*Micrococcus luteus*, *S. aureus*, *P. aeruginosa*, *E. coli*) and fungal (*Aspergillus fumigatus*, *Aspergillus niger*) strains [58]. While bacteria are resistant to DAPT, DAPT-AuNPs displayed differential antibacterial activity against the Gram-negative bacteria. Furthermore, conjugation of non-antibiotic drugs (e.g., guanidine, metformin, 1-(3-chlorophenyl)biguanide, chloroquine diphosphate, acetylcholine chloride, and melamine) as co-ligands with DAPT on AuNPs exerted non-selective antibacterial activity and a two–fourfold increased activity against Gram-negative bacteria [13]. When used in vivo, orally ingested DAPT-AuNPs showed better protection by increasing the intestinal microflora in *E. coli*-infected mice. After 4 weeks of treatment, the DAPT-AuNPs cleared the *E. coli* infection with no sign of mitochondrial damage, inflammation (increase in *firmicutes*) or metabolic disorders (reduction in *bacteroidetes*) in the mice [112].

The virucidal effects of the AuNP-based systems have been reported against several infectious diseases caused by influenza, measles [154], dengue [155, 156] and human immunodeficiency [115] viruses. Their anti-viral activity was attributed to the ability of AuNPs to either deliver anti-viral agents, or the ability to transform inactive molecules into virucidal agents [154, 156]. AuNPs synthesized using garlic water extracts inhibited measles viral growth in Vero cells infected with the measles virus. When the cells were exposed to both the virus and AuNPs at the same time, they blocked infection of Vero cells by the measles virus [154]. The AuNPs were nontoxic to the Vero cells up to a concentration of 100 µg/mL but inhibited viral uptake by 50% within 15–30 min at a concentration of 8.8 μg/mL [154]. Based on the Plaque Formation Unit assay, the viral load was reduced by 92% after 6 h exposure to 8.8 μg/mL of the AuNPs. The AuNPs interacted with the virus directly and blocked its transmission into the cells [154]. Modification of the AuNP surface with ligands that bind to the virus [156] or anti-viral agents [115, 155] protected them from degradation, enhanced their uptake and delivery onto the cells. The charge of the AuNPs also played a role, with cationic AuNPs being more effective in the delivery and efficacy of the AuNPs than the anionic and neutrally charged NPs. Cationic AuNPs complexed with siRNA inhibited dengue virus-2 replication in dengue virus-2-infected Vero and HepG-2 cells and also the virus infection following pre-treatment of the virus with AuNPs [155]. Inactive molecules are transformed into highly potent anti-viral agents after conjugation to AuNPs. One such example is the transformation of SDC-1721 peptide, a derivative of TAK-779, which is an antagonist of CCR5 and CXCR3 receptors for HIV-1 strain. SDC-1721 has no activity against the HIV-1, but when conjugated to the AuNPs it inhibited HIV-1 infection of the human phytohemagglutinin-stimulated peripheral blood mononuclear cells. The inhibitory effects of SDC-1721-AuNPs were comparable to the TAK-779 [115].

#### AuNPs as PT Agents

Diseased cells are sensitive to temperatures above 40 °C; cancer cells in particular appear to be even more sensitive to these high temperatures. Studies have shown that high fevers in cancer patients either reduced the symptoms of cancer or completely eradicated the tumors as a result of erysipelas infections [33, 157, 158]. Historically, fevers induced by bacterial infections, hot desert sand bath, or hot baths were used to increase the body temperature in order to kill the cancer cells [157]. These findings gave birth to PT therapy (PTT), which is mostly used for the treatment of cancer. PTT makes use of organic photosensitizers (indocyanine green, phthalocyanine, heptamethine cyanine) that are irradiated by the external source to generate heat energy that will increase the temperature to 40–45 °C (hyperthermia) in the target cells. Hyperthermia then triggers a chain of events (such as cell lysis, denaturation of the genetic materials and proteins), resulting in the destruction of the diseased cells [57, 158–160].

The organic dyes are used alone, or in combination with chemotherapy and radiotherapy for enhanced efficacy [157, 160]. Ideally, the effects of the PT agents must be confined to target cells and display minimal bystander effects. However, the organic PT dyes have several limitations such as toxic bystander effects, susceptibility to photobleaching and biodegradation [159]. In recent years, AuNPs are being explored as alternative PT agents as they exhibit strong plasmonic PT properties, and depending on their shape, they can absorb visible or NIR light. Absorption of light in the NIR spectrum is an added advantage that can allow deep tissue PTT [158, 161, 162]. Unlike organic dyes, AuNPs operate in an optical window where the absorption of light by interfering biological PT agents such as hemoglobin, melanin, cytochromes and water is very low [158, 161, 162].

The practicality of AuNP-based PTT has been demonstrated through in vitro and in vivo studies [158, 162, 163]. When the AuNPs are exposed to light, they can convert the absorbed light energy into thermal energy within picoseconds [57, 158, 159], consequently activating cell death via necrosis or apoptosis in the target cells or tissues. AuNP-based hyperthermia in diseased cells has been reported to occur at half the amount of the energy required to kill normal cells, thus perceived to be safer and better PT agents than the conventional dyes [33, 160]. AuNPs can be easily modified to have localized and enhanced PT activity by targeting and accumulating in only diseased cells through either active or passive targeting. And since the tumor environment is already hypoxic, acidic, nutrient starved and have leaky vasculature, the tumors will be most sensitive to the AuNP-based hyperthermia than the surrounding healthy cells and tissues [33, 160].

AuNP-based PTT has been extensively studied [158, 161, 162] and established that AuNPs (e.g., AuNRs, nanocages and nanoshells) that absorb light in the NIR spectrum are best for in vivo and deep tissue PTT [161]. While the ones that absorb and emit light in the visible spectrum (AuNSs and hollow AuNPs) have been demonstrated to treat diseases that affect shallow tissues (up to a depth of 1 mm), which could be of benefit to superficial tumors [158, 161, 162], ocular surgery [164, 165], focal therapy and vocal cord surgery [158, 165]. Although the PTT effects of AuNSs are limited in vivo or for use in deep tissues, combination therapy or active targeting can be incorporated to facilitate target-specific effects [158, 161, 163]. The AuNPs in the combination therapy will serve dual functions as both drug sensitizer and a PT agent, and was shown to enhance anticancer effects of chemotherapeutic drugs [158, 162, 163]. AuNS-Dox combination demonstrated enhanced cancer cell death after laser exposure when compared to the individual effects of the AuNSs and Dox with and without laser treatment [158].

**Fig. 9:**
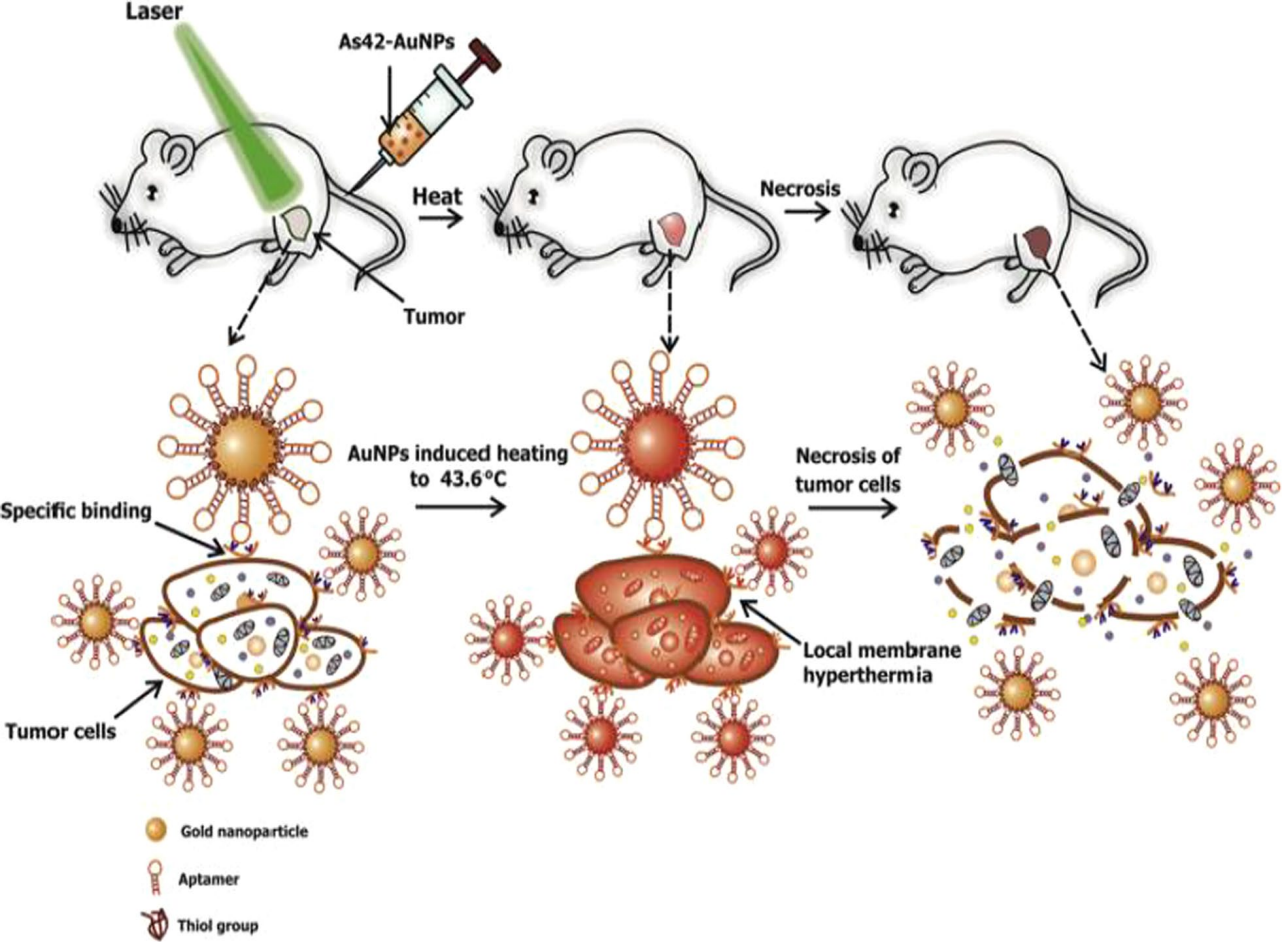
*In vivo* plasmonic PT therapy of cancer cells using targeted AuNSs. As42-AuNPs localized in HSPA8-expressing tumor cells after i.v injection. Exposure to laser treatment resulted in hyperthermia that caused cancer cell death. Reproduced with permission [163]. Copyright 2017, Elsevier

Active targeting on its own can also improve AuNP uptake, localization and target-specific PT effects, which can be viewed in real time by adding fluorophores. AuNSs (25 nm) loaded with transferrin targeting molecules and FITC were shown to accumulate and destroy human breast cancer cells at a higher rate than in non-cancer cells and had better efficacy than the untargeted AuNSs [57]. An independent study also demonstrated that DNA aptamers (As42)-loaded AuNSs (As42-AuNP) induced selective necrosis in Ehrlich carcinoma cells that express HSPA8 protein, a receptor for the aptamers. None of these effects were observed in blood and liver cells mixed with target cells, or cells treated with the AuNSs without laser treatment [163]. The PT effects of the As42-AuNP were replicated in mice transplanted with Ehrlich carcinoma cells in their right leg. As shown in Fig. 9, tail-vein injections of As42-AuNPs followed by laser irradiation resulted in targeted PT destruction of the cancer cells. The As42-AuNPs reduced tumor size in a time-dependent manner; cell death was attributed to increased temperature up to 46 °C at the tumor site. The tumor in mice treated with As42-AuNPs without laser treatment and the AuNPs conjugated with nonspecific DNA oligonucleotide continued to grow but at the lower rate compared to mice injected with PBS. This suggests that the AuNPs were also localized in the tumor [163]. In cases where AuNSs are not efficient for deep tissue PTT, other shapes such as nanocages, nanoshells and AuNRs can be used [158]. Alternately, the visible light absorption of the AuNSs can be shifted to NIR by using processes such as two-photon excitation [57].

**Fig. 10:**
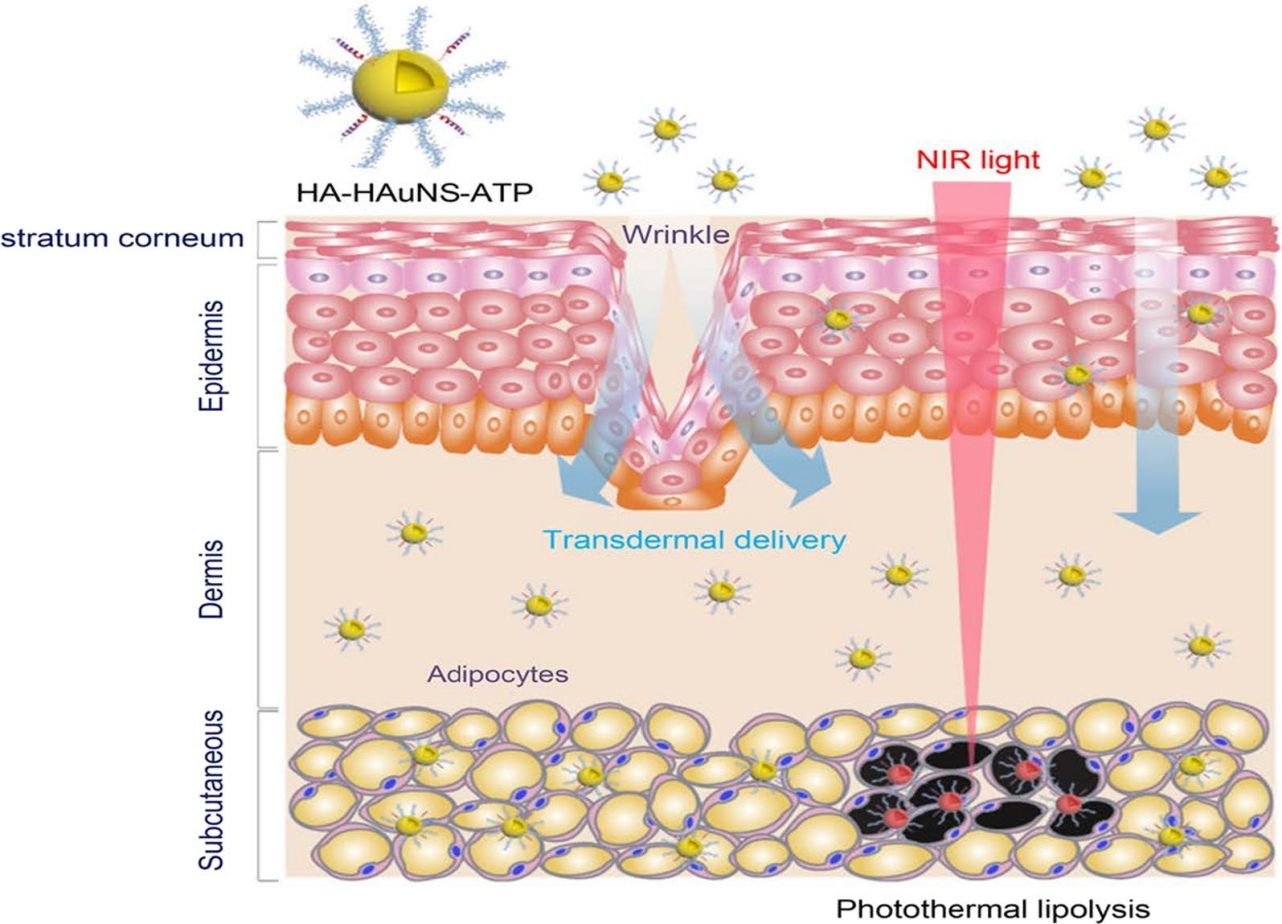
PT lipolysis of the sWATs using HA-HAuNS-ATP. The ATP was conjugated to the AuNSs for targeted delivery and destruction of the prohibitin-expressing sWATs after NIR laser exposure. Reproduced with permission [52]. Copyright 2017, American Chemical Society

The PT effects of the AuNPs have also been reported for the reversal of obesity [52, 56], using hollow AuNSs (HAuNSs) [52] and AuNRs [56] for the PT lipolysis of the subcutaneous white adipose tissue (sWAT) in obese animals. The HAuNSs were modified with hyaluronate and adipocyte targeting peptide (ATP) to produce HA–HAuNS–ATP conjugate [52]. Hyaluronate was used to ensure topical entry of the HA–HAuNS–ATP through the skin [52, 166], while ATP will recognize and bind to prohibitin once the HAuNSs are internalized. Prohibitin is a receptor that is differentially expressed by the endothelial cells found in the WAT vasculature of obese subjects [5, 52, 55]. The HA–HAuNS–ATP was topically applied in the abdominal region of the obese mice, and through hyaluronate were transdermally shuttled through the epidermis into the dermis where the ATP located the sWATs (Fig. 10**)**. Illumination of the target site with the NIR laser selectively induced PT lipolysis of the sWAT in the obese mice and reduced their body weight [52]. The AuNRs were used in the photothermolysis-assisted liposuction of the sWATs in Yucatan mini pigs. The untargeted PEG-coated AuNRs (termed NanoLipo) were injected in the sWATs through an incision, followed by laser illumination to heat up the sWATs, which was then aspirated using liposuction. The amount of fat removed from NanoLipo-treated porcine was more than the one removed with conventional suction-assisted lipectomy (SAL). NanoLipo-assisted fat removal had several advantages over the conventional SAL; it took less time (4 min) for liposuction compared to 10 min for SAL, the swelling in the treated site healed faster, and the weight loss effects lasted over 3 months post-liposuction [56].

AuNP-based PTT clearly offers a lot of advantages compared to the conventional agents. Their biocompatibility allows for broader applications both in vitro and in vivo. Moreover, they can be customized based on their shapes for shallow (AuNSs) [158, 161, 162] or deep tissue (AuNRs and stars) PTT [158, 161]. At 1–100 nm diameter, AuNPs and its conjugates can circulate long enough to reach and accumulate in the target tissues, with or without targeting moieties [159, 167]. Active targeting can be used to ensure localized PT effects through various routes of administration and might be effective for solid and systemic diseases. AuNP-based PTT can also be used to sensitize cancer cells when administered in combination with chemotherapy, gene therapy and immunotherapy [159]. Therefore, AuNP-based PTT has potential for treatment of chronic diseases [161].

## Toxicity of AuNPs

AuNPs can play an important role in medicine, as demonstrated by the preclinical and clinical studies under review. Their full potential in clinical application as both diagnostic and therapeutic agents can only be realized if they do not pose any health and environmental hazards. While their use in vitro appears to be inconsequential, in vivo application can be hampered by their potential toxicity, which could be detrimental to human health. A major concern with their clinical use is that AuNPs are non-biodegradable and their fate in biological systems has not been fully studied [5, 30]. Although AuNPs are considered to be bio-inert and compatible, their properties (size, shape, charge and composition) raise concerns as they can alter their pharmacokinetics when used in biological environment [27, 34, 118]. The toxicity of AuNPs of varying sizes and shapes has been demonstrated in animals [27, 118]. These NPs can accumulate in the RES organs where they induce damage.

AuNPs are 1–100 nm in diameter which makes them smaller than most of the cellular components. At these sizes, AuNPs can passively transverse cellular barriers and blood vessels by taking advantage of the EPR effect in pathological cells. AuNPs with smaller diameters (1–2 nm) can easily penetrate cell membranes and biologically important cellular organelles such as mitochondria and nuclei [7, 168]. Accumulation of AuNPs in these organelles induces irreversible damage that can cause cellular demise. On the contrary, AuNPs larger than 15 nm are restricted to the cytoplasmic spaces and unable to penetrate internal organelles [168]. These features are desirable for targeting pathological cells, however, AuNPs can also be taken up by healthy cells and alter their physiology [118]. Administration of AuNP-based therapeutics can be done via different routes (i.e., intranasal, oral, transdermal, i.p or i.v) and transported through blood vessels into different tissues and organs [34, 118]. They are able to pass through the blood brain barrier and the placental barrier [34]. Toxicity is size dependent, with certain sizes of AuNPs being well tolerated, while others could be lethal to healthy tissues. Unfunctionalized AuNSs at 8, 17, 12, 37 nm caused physical changes (i.e., change the fur color, loss of bodyweight, camel-like back and crooked spine) within 14 days of treatment (2 doses of 8 mg/kg/week) in rats [118]. Most (> 50%) of the rats died within 21 days (i.e., after 3 doses), and abnormalities in the RES organs (liver, lungs and spleen) were observed. On the contrary, mice treated with 3, 5, 50 and 100 nm AuNPs were not affected by the NPs and no adverse effects or death occurred throughout the duration (50 days) of the study [118]. In diet-induced obese rats that received i.v injections of 14 nm cAuNPs, the NPs were detected in various tissues after 24 h and were mostly confined to the RES organs [55].

The shape, charge and surface chemistry of AuNPs can influence their toxicity. These factors can determine how AuNPs will interact with the biological systems, their cellular uptake and effects on the cells. AuNSs are readily taken up by cells and proven to be less toxic than other shapes such as rods and stars. AuNP surfaces are charged and will influence how they interact and behave within a biological environment [169]. Cationic AuNPs are likely to be more toxic compared to neutral and anionic AuNPs, as their charge allows these NPs to easily interact with negatively charged cell membranes and biomolecules such as DNA. Both the positively and negatively charged AuNPs have been associated with mitochondrial stress, which was not observed with the neutrally charged AuNPs [34, 35].

The shell that forms on the surface of the AuNP core can also influence the functioning of the NPs. These are usually reducing and/ or stabilizing agents such as citrate and CTAB, and once subjected to a biological environment, these molecules can cause either the desorption or absorption of biomolecules found in the biological environment. This can result in the formation of a corona or cause the NPs to become unstable. Citrate- and CTAB-capped AuNPs are highly reactive, which can facilitate the attachment of biocompatible polymers such as PEG, polyvinyl-pyrrolidone, poly (acrylic acid), poly(allylamine hydrochloride), and polyvinyl-alcohol) or biomolecules such as albumin and glutathione to prevent the formation of AuNP-corona with serum proteins. These molecules serve as a stabilizing agent and form a protective layer that can mask the AuNPs from attacks by phagocytes [7, 29, 34, 170] and prevent off-target toxicity [7]. As discussed in “AuNP-Based Therapies” section, AuNPs can be functionalized with targeting and therapeutic agents to define their targets and effects [34].

In addition to their physicochemical properties, the dosage, exposure time and environmental settings also influence the activity of AuNPs. Lower doses and short-term exposure times might render AuNP as nontoxic, while increasing these parameters will lead to cytotoxic effects [34]. Moreover, in vitro studies do not always simulate in vivo studies. At times, AuNPs that seem to be nontoxic in cell culture-based experiments end up being toxic in animal experiments. Many factors could be responsible for these discrepancies [118], and some steps have been identified that can guarantee the safety of AuNPs in biomedical applications. The biocompatibility and target specificity of AuNPs can be improved by modifying the surface of the NPs. Attaching targeting moieties on the AuNPs can channel and restrict their effects to specific targets or pathological cells [5, 55, 127]. Modification of AuNP surface with bio-active peptides provides a platform for developing multifunctional AuNPs with enhanced specificity, efficacy and potentially sustainable effects [11, 127]. All of these effects will be instrumental in the design and development of AuNP-based systems for clinical applications.

## Clinical Application of AuNPs

Nanotechnology has the potential to shape the future of healthcare systems and their outcomes. Its promise of creating highly sensitive and effective nanosystems for medicine has been realized with the introduction of organic nanoformulations for cancer treatment. These systems have already paved the way for nanomaterials into clinical applications: doxil and abraxane have been in the market for over two decades and demonstrated the potential of nanotechnology in medicine [1, 2]. More recently, this technology has been used for the development of the SARS-CoV-2 lipid NP-based vaccine to fight against the COVID-19 pandemic [171]. Inorganic nanosystems such as AuNPs offer many advantages over their organic counterparts, yet few of these systems are used clinically (Table 2) [19, 32].

**Table 2:** AuNP-based formulations approved for clinical trials by FDA. Adapted from [19, 32]

AuNP formulation	Condition or disease	Properties of the metallic NPs	NCT number
Aurrimune	Late stage pancreatic, breast, colon, melanoma, sarcoma and lung cancer	27 nm AuNPs core loaded with TNF-α and PEG	NCT00356980 NCT00436410
Aurolase	Refractory and/or recurrent tumors for head and neck cancer Primary and/or metastatic lung tumors	150 nm silica-gold nanoshells coated with PEG	NCT00848042 NCT01679470
Sebacia Microparticles	Acne vulgaris	150 nm silica-gold nanoshells coated with PEG	NCT02219074 NCT02217228
C19-A3 GNP peptide	Type 1 diabetes	AuNPs with peptide fragment related to insulin	NCT02837094
NU-0129	Gliosarcoma		
Recurrent glioblastoma	AuNPs with nucleic acid	NCT03020017	
NANOM-FIM	Stable angina Heart failure Atherosclerosis		
Multivessel coronary artery disease	Silica- AuNPs vs AuNPs with silica–iron oxide shells with photothermic burning or melting effect onto the lesion	NCT01270139	
Nano Care Gold	Cavity pre-treatment in Caries class Ii	AgNPs and AuNPs in 70% isopropyl alcohol	NCT03669224
Exhaled Breath Olfactory Signature (Artificial Nose)	Pulmonary hypertension	AuNPs coated with organic ligands as sensor array for detection of volatile organic compounds in breath of patients	NCT02782026
Na-nose	Gastric diseases	Functionalized AuNPs and carbon nanotubes nanosensor arrays	NCT01420588
Nanomedical Artificial Olfactory System	Parkinson’s disease Parkinsonism	Functionalized AuNPs and carbon nanotubes nanosensor arrays	NCT01246336

While several AuNP-based drugs are some of the inorganic nanomaterial-based drugs that were tested in clinical trials, they are not progressing at the same rate as organic liposome-based nanodrugs. Aurimune (CYT-6091) and aurolase were the first of AuNP-based formulations to undergo human clinical trials for the treatment of solid tumors. CYT-6091 clinical trials started in 2005 for delivery of recombinant TNF-α as an anticancer therapy in late-stage pancreatic, breast, colon, melanoma, sarcoma and lung cancer patients. CYT-6091 consists of 27-nm cAuNPs loaded with TNF-α and thiolated PEG. The CYT-6091 nanodrug has achieved safety and targeted biologic response at the tumor site at a dose lower than that required for TNF-α alone [16, 17]. CYT-6091 is approved and yet to start phase II clinical trials in combination with chemotherapy. Based on phase II clinical trial strategy, several variants of CYT-6091 have been developed and tested in preclinical studies. All the nanosystems contain TNF-α with either chemotherapy (paclitaxel, dox and gemcitabine), immunotherapy (Interferon gamma) or apoptosis inducing agents attached to the 27 nm cAuNPs [14–16]. The AuNP conjugates preferentially accumulated in the tumor sites after systemic administration through the EPR effect and vascular targeting effects of the TNF-α. The AuNPs were not detected in the healthy tissues, and the anti-tumor effects of TNF-α were restricted to the tumor environment [14, 16, 19].

The first clinical trial for the PT treatment with AuroLase® for refractory and/or recurrent head and neck cancers was completed. Information on the outcome of this trial is still pending. The second trial is set to evaluate the effects of AuroLase® on primary and/or metastatic lung tumors in patients where the airway is obstructed [19]. The number of human trials based on AuNP-based formulation is increasing, covering the treatment of a wide range of medical conditions including skin, oral, heart and neurological diseases. AuNP-formulation (150 nm silica-gold nanoshells coated with PEG), which is similar to AuroLase®, was approved for PT treatment of moderate-to-severe inflammatory acne vulgaris. The nanoshells were topically applied on the acne area and transdermally delivered into the follicles and sebaceous ducts through low-frequency ultrasound or massage. Nanoshells applied through massage were effective in penetrating the shallow skin infundibulum (90%) and the sebaceous gland (20%), while the low-frequency ultrasound can penetrate both shallow and deep skin tissues. NIR laser treatment resulted in focal thermolysis of the sebaceous glands in the affected area and disappearance of the acne [18, 167]. The gold–silica nanoshells were well-tolerated, showed no systemic toxic effects with minor side effects (reddiness and swelling) at the treatment site [18]. AuNPs offer many health benefits based on their unique properties but at the same time have raised a lot of political and ethical issues, and resulted in termination of some clinical studies (NCT01436123).

## Conclusion and Future Perspectives

Applications of AuNPs in biomedicine are endorsed by their unique physicochemical properties and have shown great promise as theranostic agents. The increasing interest in biomedical applications of AuNPs is further encouraged by the biocompatibility and medical history of bulk gold, which suggests that the gold core in AuNPs will essentially display similar or improved properties [3]. But at the same time their small size can infer unique properties that will completely change their pharmacokinetics [144]. The diverse biomedical applications of AuNPs in diagnostics and therapeutics herein discussed demonstrate their potential to serve as adjunct theranostic agents. They can be used as drug delivery, PTT, diagnostic and molecular imaging agents [12, 33, 128]. In time, and with better knowledge of mechanisms of action, more AuNP-based systems will obtain approval for clinical use. However, the excitement of these biomedical applications of AuNPs should unequivocally be balanced with testing and validation of their safety in living systems before any clinical applications.

In conclusion, more work needs to be done to taper the toxicity of AuNPs. This can be achieved by introducing biocompatible molecules on their surface [14, 15, 58, 159], and developing new and better synthesis methods, such as the use of green chemistry to produce biogenic NPs. All these developments may further broaden the applications of AuNPs in nanomedicine. AuNPs are non-biodegradable, and off-target distribution could result in chronic and lethal effects. All these concerns must be addressed before clinical translation; the existing trials will soon provide some clarity on their impact in human health. Should their health benefits outweigh their potential risks as is the case with the existing clinical drugs, it is a matter of time before they are approved for clinical use.

## Data Availability

All the information in this paper was obtained from the studies that are already published and referenced accordingly.

## Abbreviations

5-FU: 5-Fluorouracil
AD: Alzheimer's disease
ADDLs: Amyloid-beta-derived diffusible ligands
ASOs: Antisense oligonucleotides
AuNPs: Gold nanoparticles
AuNPsQ: Quantum-sized AuNPs
AuNRs: Gold nanorods
AuNSs: Gold nanospheres
BCA: Bio-barcoding assay
cAuNPs: Citrate-capped AuNPs
COVID-19: Corona virus disease 2019
CSF: Cerebrospinal fluid
CT: Computed tomography
CTAB: Cetyltrimethylammonium bromide
DABCYL: 4-((4′-(Dimethyl-amino)-phenyl)-azo)benzoic acid
DAPT: 4,6-Diamino-2-pyrimidinethiol
Dox: Doxorubicin
EDC: 1-Ethyl-3-(3-dimethylaminopropyl) carbodiimide
EPR: Enhanced permeability and retention
FA: Folate
FDA: Food and Drug Administration
FITC: Fluorescein isothiocyanate
FRET: Fluorescence resonance energy transfer
HAuNSs: Hollow AuNSs
HF: High-fat
HU^−1^: Hounsfield unit
LFAs: Lateral flow assays
LSPR: Localized surface plasmon resonance
MDR: Multidrug resistant
MGF: Mangiferin
MPA: Mercaptopropionic acid
MWPLP: Microwave-induced plasma-in-liquid process
NCL: Nucleolin
NIR: Near-infrared
NSMs: Nanostructured materials
*P. jirovecii*: *Pneumocystis jirovecii*
PEG: Polyethylene glycol
PSA: Prostate-specific antigen
PSMA: Prostate-specific membrane antigen
PT: Photothermal
PTT: Photothermal therapy
QDs: Quantum dots
RES: Reticuloendothelial system
ROS: Reactive oxygen species
SARS-CoV-2: Severe acute respiratory syndrome-coronavirus-2
SCID: Severe combined immunodeficiency
SPR: Surface plasmon resonance
sWAT: Subcutaneous white adipose tissue
TOAB: Tetrabutylammonium bromide
TRMs: Tissue-resident macrophages
VEGF: Vascular endothelial growth factor
WAT: White adipose tissue
